# Dynamic Bayesian networks for neural information flow: evaluation of continuous and discrete scoring metrics

**DOI:** 10.1007/s10827-026-00945-6

**Published:** 2026-07-28

**Authors:** Jacob Thomas-Hegarty, Stefan R. Pulver, V Anne Smith

**Affiliations:** 1 School of Biology, University of St Andrews, St Andrews, Scotland; 2 School of Psychology and Neuroscience, University of St Andrews, St Andrews, Scotland; 3 Institute of Behavioural and Neural Sciences, University of St Andrews, St Andrews, Scotland

**Keywords:** Neural information flow, Dynamic Bayesian network, Structure learning, Granger causality

## Abstract

Neural information flow describes the movement of activity between neurons or brain areas. Advances in experimental methods have allowed production of large amounts of observational data related to neuronal activity from the single-neuron to population level. Most current methods for analysing these data are based on pairwise comparison of activity, and fall short of reliably extracting neural information flow network structure. Dynamic Bayesian networks may overcome some of these limitations. Here we evaluate the performance of a range of Bayesian network scoring metrics against the performance of multivariate Granger causality and LASSO regression for their ability to learn the connectivity underlying simulated single-neuron and neuronal population data. We find that discrete dynamic Bayesian networks are the best performing method for single-neuron data, and perform consistently for neural-population data. Continuous dynamic Bayesian networks have a tendency to learn overly dense structures for both data types, but may have utility in scoping studies on single-neuron data. Multivariate Granger causality is the most robust method for learning structure of neural information flow between neural-populations, but performs poorly on single-neuron data. Significance testing within multivariate Granger causality produces variable results between data types. Overall, this work highlights how the analysis of neural information flow can vary depending on the type and structure of underlying data, and promotes discrete dynamic Bayesian networks as a useful and consistent tool for neural information flow analysis.

**Supplementary Information:**

The online version contains supplementary material available at https://doi.org/10.1007/s10827-026-00945-6.

## Introduction

Neural information flow (NIF) refers to the flow of activity across brain structures in different behavioural and cognitive states. Understanding how NIF structures change during different behaviours could improve our understanding of how underlying circuits function. For example, in the *ex vivo* *Drosophila* larval ventral nerve chord, distinct network activity modes are associated with different motor behaviour (Pulver et al., 2015). Understanding the differences in information flow between these network modes may aid in understanding of how these behaviours are brought about by the same anatomical structures. Similarly, pathology linked changes in functional connectivity have been observed in Alzheimer’s disease patients (Yu et al., 2021). Understanding how this relates to changes in NIF network structures may reveal functional changes that help target novel interventions and improve diagnostics.

Although it constrains NIF, structural connectivity cannot necessarily be used to infer NIF network structure, with some of the strongest structural connections not necessarily providing the strongest pathways of information flow (Benozzo et al., 2024). NIF analysis requires simultaneous observation of neuronal activity in different regions. Such data can be collected using techniques with varying invasiveness and temporal and spatial resolution, such as functional magnetic resonance imaging (fMRI), electroencephalography (EEG), calcium imaging and microarray electrophysiology. Analyses aiming to learn connectivity structures from these types of data may fall into the category of either functional or effective connectivity. Functional connectivity describes statistical associations between activity patterns in brain areas. Effective connectivity attempts to uncover the causal interactions between these units, and generally requires testing of an *a priori* causal neuronal structure, making structure learning difficult (Valdes-Sosa et al., 2011). However, likely due to misunderstandings surrounding causal influence, many methods are mislabelled as producing effective connectivity even when they do not uncover causal interactions. As such, these labels have limited utility without understanding a method’s individual assumptions and aims. NIF structures must represent directional and direct connectivity between brain regions. In other words, the network structure must define the direction of flow of influence between regions, and not include any direct connections between regions whose connectivity is mediated by another observed region. This means that, although not necessarily representing causal structures, NIF presents a more thorough approach to understanding activity patterns than many functional connectivity methods, perhaps providing candidate models for future causal analysis.

Currently, when attempting to map influence between neurons or brain areas it is common to analyse neuronal activity in a pairwise manner, comparing lagged or un-lagged activity of each combination of brain areas, and deeming them connected if this similarity is significant (Wang et al., 2021). These methods fall short of reliable inference of NIF structures for a number of reasons. All un-lagged methods, such as correlation analyses, are unable to infer direction of connectivity between brain regions. Some methods utilise temporal information to infer the direction of influence between a pair of brain areas. Granger causality (GC) (Granger, 1969; Geweke, 1982, 1984) and its frequency domain counterpart, partial directed coherence (Baccalá & Sameshima, 2001), are commonly used examples of such methods (Shojaie & Fox, 2022; Seth et al., 2015). Another commonly used method, transfer entropy, is a model free version of the classical GC framework. For Gaussian variables, transfer entropy and GC are equivalent (Barnett et al., 2009). Built upon the intuition that a cause must precede its effects, GC quantifies whether the past of a potential parent variable improves prediction on the future of a potential child variable, given the past of the child. However, formation of network structures from individual pairwise comparison often leads to mis-identification of spurious connections between brain regions in the cases of indirect connectivity, common innervation or common sensory input (Novelli & Lizier, 2021). Multivariate Granger causality (MVGC) attempts to allay these concerns through quantification of the improvement in prediction of the future activity pattern of one variable when information on the past of another is included conditioned on the past of all other variables in the system (Geweke, 1984). However, MVGC may still lead to the learning of spurious connections, for example in the case of collider structures in the data generating process or confounding by unobserved variables (Eichler, 2005).

A further limitation of pairwise methods is the arbitrary nature of choosing a threshold on some metric whereby a connection is deemed present in the network, complicating interpretation of results. Significance testing on these metrics may be employed; however, in large networks stringent corrections for multiple testing must be performed, potentially reducing statistical power and increasing occurrence of false negatives (Higgins et al., 2019). Analytical methodologies which learn structures of NIF at the network level, as opposed to interaction level, may provide increased reliability and interpretability of findings. Such methods would be capable of linking entire NIF network structures to behaviour without the need for qualitative threshold selection.

A Bayesian network (BN) is a graphical representation and set of parameters describing the dependence and conditional independence relationships, represented as edges, between a group of variables, represented as nodes. Directed links from a parent to child node represent the statistical dependence of the child on the parent. Each node is conditionally independent of all its non-descendants given its parent nodes. Therefore a BN can map unmediated information flow between its nodes. BN structures can be learned at the network level, without utilising pairwise comparison of nodes’ activity. These models have been utilised widely in a neuroscience context, particularly in fMRI data (Bielza & Larraeñaga, 2014). Here they have uncovered alterations in NIF associated with mild cognitive impairment (Li et al., 2013) and breast cancer treatment (Phillips et al., 2022). However, BN structures may have mathematically equivalent counterparts where the direction of some links are reversed, making interpretation of directionality in such structures problematic. Further, BNs are unable to represent cyclic structures, which is problematic in a NIF context due to the abundance of reciprocal connections within nervous systems (Lin et al., 2024; Edelman & Gally, 2013). These issues limit the utility of static BNs for NIF structure learning.

Through inclusion of temporal information, dynamic BNs (DBNs) overcome these limitations. DBNs have shown strong performance relative to current methods in learning ground-truth connectivity structure in simulated fMRI (Liu et al., 2019; Rajapakse & Zhou, 2007) and spiking neuronal data, particularly when connections are simulated using highly non-linear synaptic integration (Eldawlatly et al., 2008, 2010). NIF structures have been learned as DBNs from a range of data modalities and research areas. Utilising fMRI data, DBN representations of NIF have been used to study changes associated with dementia (Burge et al., 2007; Liu et al., 2019), Parkinson’s disease (Li et al., 2006), mild cognitive impairment (Ramakrishna & Ramasangu, 2019), schizophrenia (Kim et al., 2008), addiction (Zhang et al., 2005), ageing (Dang et al., 2018) and various cognitive and behavioural states (Rajapakse & Zhou, 2007; Rajapakse et al., 2008; Wu et al., 2013; Warnick et al., 2017). Similar methods have been used with EEG (Mutlu & Aviyente, 2009), electrocorticography (Zhang et al., 2010), and local field potential data (Tsukahara et al., 2022; Xing et al., 2025; Smith et al., 2006) to study NIF related to schizophrenia, hand movement, social gaze, seizures, and auditory circuits.

There are a variety of methodologies for learning the structure of DBNs. Constraint-based methods use conditional independence tests to learn network structure. Alternatively, score based methods search heuristically for the optimal network, utilising scores which aim to quantify the probability of a given DBN structure given the observed data. Score based methods are more widely utilised and may be more accurate (Scutari et al., 2019). There are a variety of scores for learning network structures from either discrete or continuous Gaussian data. Although the former is most commonly used, information lost through discretisation and the summative nature of neuronal influence may mean that continuous scores are advantageous on neuronal data. There is also evidence that they may be more robust to noise on simulated fMRI data than their discrete counterpart (Wu et al., 2013).

The structure of data across spatial scales in neuroscience differs dramatically, from highly non-linear single-neuron dynamics, to more linear oscillatory neuronal population data. Understanding how performance of NIF structure learning methods differ dependent on this context is vital when selecting appropriate analysis tools for a given data type. This may be particularly relevant for continuous DBNs given their assumption of linearity in data streams, but the impacts of these violations are unknown.

Despite some examples of their use in the literature, large scale evaluation of the comparative performance of DBN scores for NIF structure learning from different data types has not been performed, leaving uncertainty around their relative performance with different modalities. This study provides an initial evaluation of range of score based metrics for continuous and discrete DBN structure learning in terms of their ability to reconstruct the connectivity of simulated single-neuron and neural-population data. For comparison, MVGC was also tested, as a frequently used method capable of learning NIF structures in a pairwise manner (Camps-Valls et al., 2023; Seth et al., 2015). Additionally, an unlagged regularized linear regression based method is included for a reference of the performance of unlagged multivariate methods.

## Methods

**Fig. 1:**
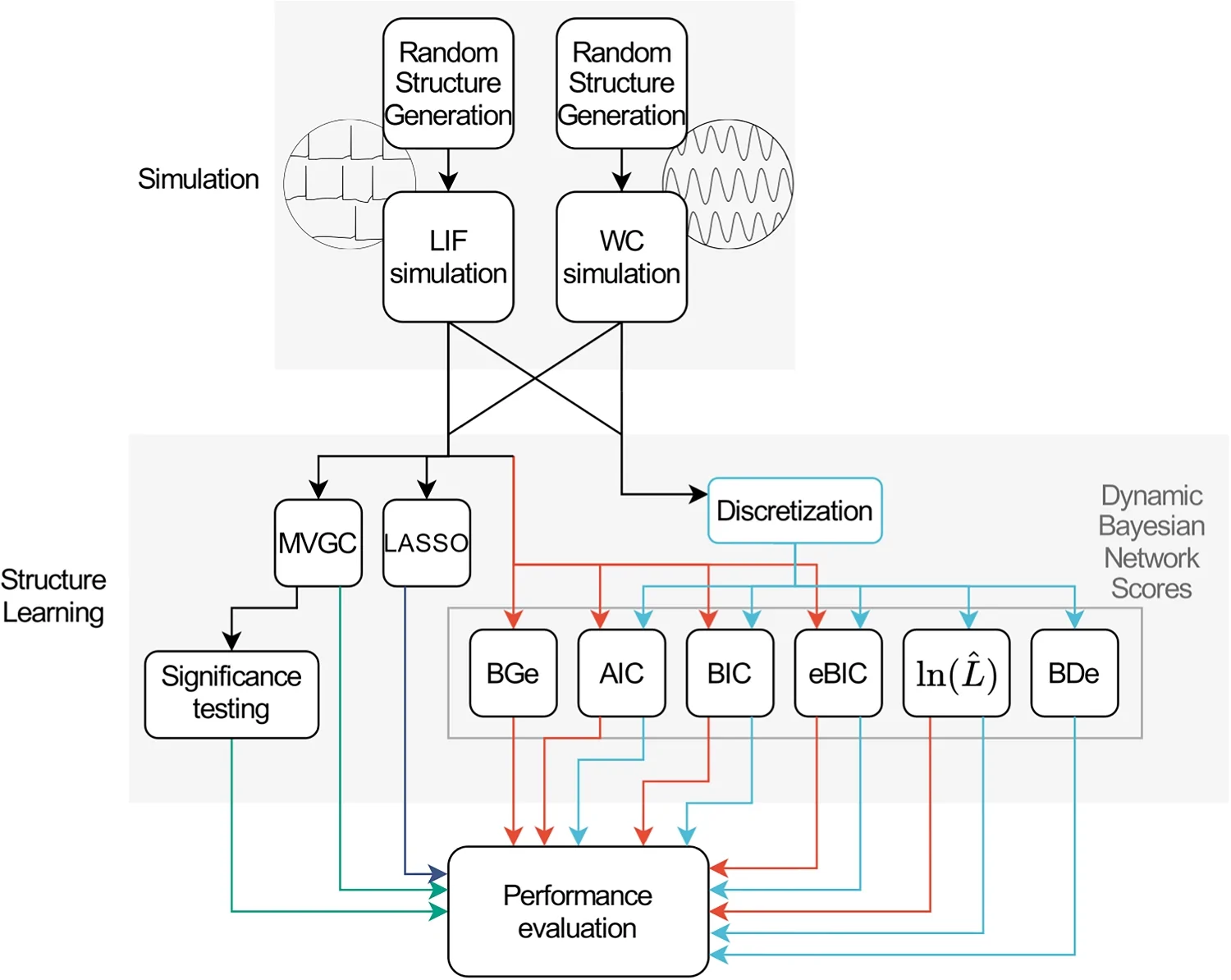
Study workflow. Two sets of randomly generated connectivity structures were generated. Activity across one set was simulated using leaky integrate and fire dynamics, and the other using a Wilson-Cowan based model of connected oscillators. Data from these simulations were used to test a range of dynamic Bayesian network learning scores, multivariate Granger causality (MVGC) and LASSO regression based methods for their ability to re-learn the structures underlying the simulated data. Where possible, the methods were tested on both discrete and continuous data. (BGe: Bayesian Gaussian equivalent, AIC: Akaike information criterion, BIC: Bayesian information criterion, eBIC: extended Bayesian information criterion, $\ln (\hat{L})$ : log-likelihood, BDe: Bayesian Dirichlet equivalent)

This study utilises two distinct simulation types to produce two datasets containing 100 in silico single-neuron and neural-population activity patterns for each node in networks of pseudo-random known structure. Each structure learning method discussed below was then used to predict the structure underlying each simulation, and findings were evaluated against the known ground-truth structure. The performance of each structure learning method was considered based on its average precision and recall, and evaluated against chance using hypergeometric testing. Figure 1 summarises this workflow.

### Simulated datasets

In order to evaluate the performance of structure learning methods, data with known underlying structure is required. Here we utilise two simulated datasets of 100 pseudo-randomly generated 10 node structures. The first dataset was produced using leaky integrate and fire equations, modelling single-neuron dynamics. The second was produced using a neural mass model, modelling the dynamics of populations of neurons. All network generation and simulation was performed in Python (Version 3.13.12).

#### Single-neuron simulation

Leaky integrate and fire (LIF) equations were used to model single-neuron membrane potentials. Change in sub-threshold membrane potential ($dV_k$) was governed by integration of synaptic inputs with exponential decay towards the resting potential $V_\text {rest}$ at rate $\tau _m$:

1 $$\begin{aligned} dV_k = \dfrac{dt}{\tau _m}\left( V_\text {rest}-V_k(t) +\dfrac{\alpha _k(t)}{g_m}\right) \end{aligned}$$

Here $V_k(t)$ is the membrane potential of neuron *k* at time-point *t*, $\alpha _k(t)$ is the total synaptic input to neuron *k* at time-point *t*, $g_m$ is a fixed parameter describing the resting membrane conductance, and *dt* is the simulation time-step. When a neuron’s membrane potential ($V_k$) reached a threshold ($V_{th}$), a spike was initiated, and the refractory counter $R_k$ set to $\tau _\text {ref}$. At the time-point immediately following this, $V_k$ was set to a firing potential $V_\text {spike}$, representing the depolarisation phase of an action potential. Following this, for the remainder of the refractory period $\tau _\text {ref}$, $V_k$ was set to $V_\text {rest}$ :

2 $$\begin{aligned} V_k(t+1)&= {\left\{ \begin{array}{ll} V_\text {spike}, & V_k(t) \ge V_{\text {th}}, \\ V_\text {rest}, & R_k(t)> 0, \\ V_k(t) + dV_k, & \text {otherwise}. \end{array}\right. } \\ R_k(t+1)&= {\left\{ \begin{array}{ll} \tau _{\text {ref}}, & V_k(t) \ge V_{\text {th}}, \\ R_k(t) - dt, & R_k(t) > 0, \\ 0, & \text {otherwise}, \end{array}\right. }\nonumber \end{aligned}$$

Networks were formed of 10 neurons. Each neuron sent four connections to different randomly selected neurons, with uniform probabilities of any non-self connection being present. Three neurons were randomly selected to be inhibitory. This level of inhibition was selected to prioritise network stability across a large range of random structures. A total of 100 structures were simulated.

Synaptic input *α * to each neuron *k* at time-step *t* was modelled using conductance based synapses:

3 $$\begin{aligned} \alpha _k(t)&= g_k^{\text {ex}}(t)(E_\text {ex} - V_k(t)) \; + \\&\quad \,\,\,\, g_k^\text {inh}(t)(E_\text {inh}-V_k(t)), \nonumber \end{aligned}$$

where $g_k^{\text {ex}}(t)$ and $g_k^{\text {inh}}(t)$ are the excitatory and inhibitory synaptic conductances of neuron *k* at time-point *t*. Intuitively, when $g_k^{\text {ex}}$ or $g_k^{\text {inh}}$ are high, membrane potential in the postsynaptic neuron *k* will tend towards the relevant synaptic equilibrium potentials $E_\text {ex}$ and $E_\text {inh}$. Conductances increase by $\Delta g_\text {ex}$ and $\Delta g_\text {inh}$ when the neuron receives a spike from a connected excitatory or inhibitory neuron. Otherwise they decay exponentially, with the rate of this decay controlled by the synaptic time parameters $\tau _\text {ex}$ and $\tau _\text {inh}$:

4 $$\begin{aligned} g^{\textrm{ex}}_k(t+1)&= \begin{aligned}[t]&\left( 1 - \frac{dt}{\tau _{\textrm{ex}}}\right) g^{\textrm{ex}}_k(t) \;+\\&\Delta g_{\textrm{ex}} \left( I_k(t) + \sum _{i=1}^n \textbf{A}^{E}_{ki}\, S_i(t) \right) , \end{aligned} \\ g^{\textrm{inh}}_k(t+1)&= \begin{aligned}[t]&\left( 1 - \frac{dt}{\tau _{\textrm{inh}}}\right) g^{\textrm{inh}}_k(t) \; + \\&\Delta g_{\textrm{inh}} \left( \sum _{i=1}^n \textbf{A}^{I}_{ki}\, S_i(t) \right) . \end{aligned}\nonumber \end{aligned}$$

$\textbf{A}^E$ and $\textbf{A}^I$ represent excitatory and inhibitory connectivity matrices. *n* is the number of neurons in the network, so that $\textbf{A}^E_{ki}$ and $\textbf{A}^I_{ki}$ indicate the presence or absence of an excitatory or inhibitory connection from neuron *i* to neuron *k*. $S_i(t)$ is 1 if neuron *i* fired at time-point *t* and 0 otherwise. The summation terms count the number of connected excitatory or inhibition neurons which fired at time-point *t*. Exogenous spikes are represented by $I _k (t)$, which is 1 if there is an exogenous input spike to *k* at time *t* and 0 otherwise. Each neuron received exogenous stimulation via an independent Poisson spike train with a firing rate of 25 Hz, generated via sampling of inter-spike intervals from an exponential distribution with a mean of $\dfrac{1}{25}$, modelling input to the network from unobserved excitatory neurons.

Parameter values related to neuronal dynamics were selected based on those used in previously in the literature (Vogels & Abbott, 2005). Synaptic variables $\Delta _{g_{\text {ex}}}$ and $\Delta _{g_{\text {inh}}}$ were selected based on a parameter search, optimising these values to maximise the proportion of random networks with stable firing rates and irregular firing patterns. The latter was measured through calculation of the coefficient of variation of inter-spike intervals ($\text {CV}_\text {ISI}$). The selected values and interpretation of fixed parameters in the above equations are shown in Table 1.

Simulations were run for 100 s, with a time-step (*dt*) of 0.1 ms. Data was averaged into 1 ms bins to provide 100,000 data-points for the structure learning process.

**Table 1:** Parameters utilised in leaky integrate and fire simulations

Parameter	Description	Value
$\tau _m$	Membrane time step	20 ms
$V_\text {rest}$	Resting/reset potential	-60 mV
$g_m$	Resting membrane conductance	10 nS
$V_{th}$	Threshold potential	-50 mV
$\tau _\text {ref}$	Refractory time	5 ms
$\Delta g_\text {ex}$	Excitatory conductance change	3 nS
$\Delta g_\text {inh}$	Inhibitory conductance change	3 nS
$E_{ex}$	Excitatory synaptic equilibrium potential	0 mV
$E_{inh}$	Inhibitory synaptic equilibrium potential	-80 mV
$\tau _\text {ex}$	Excitatory synaptic time step	5 ms
$\tau _\text {inh}$	Inhibitory synaptic time step	10 ms
$V_\text {spike}$	Spiking membrane potential	30 mV
*dt*	Simulation time step	0.1 ms

#### Neural-population simulation

**Fig. 2:**
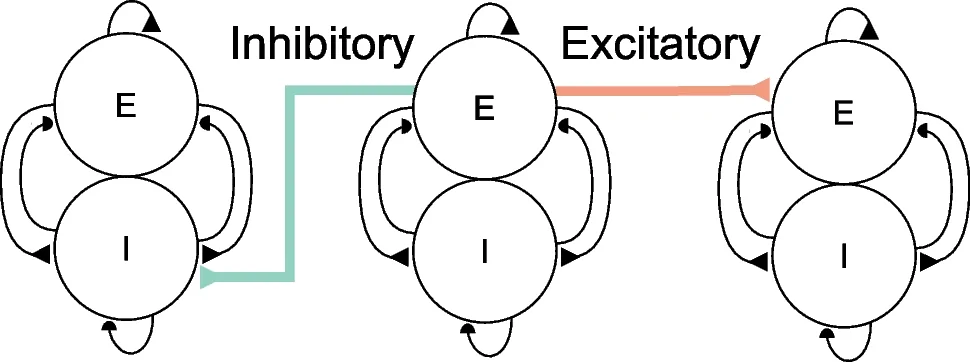
The structure of neural-populations and their exogenous connections used in neural-population simulations. One unit represents the basic structure of a Wilson Cowan oscillator, illustrating the connectivity between subpopulations. Connections between units illustrate the inter-population connectivity used to randomly connect populations in neural-population simulations. Excitatory connections between populations are represented by connecting respective excitatory subpopulations (red arrow), and inhibitory connections by connecting the excitatory subpopulation of the parent oscillator to the inhibitory subpopulation of the child oscillator (green arrow)

Wilson–Cowan oscillators are a form of neural mass model which separately model the activity of excitatory and inhibitory neurons within neuronal populations (Wilson & Cowan, 1972). These activities are generally described by two partial differential equations modelling all possible interactions between these units (Fig. 2):

5 $$\begin{aligned} \tau _E\dfrac{dE}{dt}&= -E +F(w_{EE}E -w_{IE}I+P)\\ \tau _I\dfrac{dI}{dt}&= -I +F(w_{EI}E -w_{II}I)\nonumber \end{aligned}$$

where *E* and *I* represent excitatory and inhibitory subpopulation activity, $\tau _E$ and $\tau _I$ represent the excitatory and inhibitory population time-steps, and *P* represents the exogenous excitatory input to the population. The *w* terms represent connectivity between subpopulations, with $w_{EI}$ representing the connectivity strength from the inhibitory to the excitatory subpopulation, $w_{IE}$ representing the connectivity strength from the excitatory to the inhibitory subpopulation, and $w_{EE}$ and $w_{II}$ representing the strength of endogenous connectivity within these subpopulations. In this work, the activation function *F* was a shifted sigmoidal function with threshold of *θ * and gain of *α *:

6 $$\begin{aligned} F(x;\alpha ;\theta ) = \dfrac{1}{1 + e ^{-\alpha (x - \theta )}} - \dfrac{1}{1 + e^{\alpha \theta }} \end{aligned}$$

The basic Wilson-Cowan equations were adapted to allow simulation of distinct connected neuronal populations with stochastic exogenous input in discrete time:

7 $$\begin{aligned} E_k(t+1)&= \begin{aligned}[t]&E_k(t)& \;+ \;\dfrac{dt}{\tau _E} \Bigg ( \begin{aligned}[t]&-E_k(t) \;+ F \Big ( \begin{aligned}[t]&w_{EE}E_k(t) - w_{IE}I_k(t) \; + \sum _{i=1}^n \alpha \mathbf {A^E}_{ki} E_i(t)\; + P_k(t) \Big ) \Bigg ) \end{aligned} \end{aligned} \end{aligned} \\ I_k(t+1)&= \begin{aligned}[t]&I_k(t) \;+ \dfrac{dt}{\tau _I} \Bigg ( \begin{aligned}[t]&-I_k(t)\; +F \Big ( \begin{aligned}[t]&w_{EI}E_k(t) - w_{II}I_k(t)\; + \sum _{i=1}^n \beta \mathbf {A^I}_{ki} E_i(t) \;+P_k(t) \Big ) \Bigg )\nonumber \end{aligned} \end{aligned} \end{aligned} \end{aligned}$$

where $E_k$ and $I_k$ are the excitatory and inhibitory subpopulation activity of population *k*, ${\textbf {A}}^E$ and ${\textbf {A}}^I$ are excitatory and inhibitory connectivity matrices, and *α * and *β * the excitatory and inhibitory inter-population connectivity weights. *n* is the number of neurons in the network, so that $\textbf{A}^E_{ki}$ and $\textbf{A}^I_{ki}$ indicate the presence or absence of a connection from the excitatory subpopulation of *i* to the excitatory or inhibitory population of *k* (Fig. 2), and the summation terms calculate the weighted input activity from inter-population connections. $P_k(t)$ is the exogenous input noise to population *k* at time-step *t*, modelled as independent Orstein-Uhlenbeck (OU) processes:

8 $$\begin{aligned} P_k(t+1) = P_k(t) + \theta _{OU}(\mu _{OU} - P_k (t))dt +\sigma _{OU} \sqrt{dt}\epsilon _t \end{aligned}$$

where *ε * is a random variable sampled from a normal distribution of mean 0 and variance 1. $\mu _\text {OU}$, $\theta _\text {OU}$, and $\sigma _\text {OU}$ are fixed parameters controlling the mean, rate of reversion to the mean, and volatility of the OU process respectively. The values of all fixed parameters were selected to reliably exhibit irregular peak intervals at a reasonable rate, as measured using the coefficient of variation of inter-peak intervals ($CV_{IPI}$), and number of peaks across randomly connected 10 neuron networks, and are displayed in Table 2.

Inter-population connectivity matrices were formed in a similar manner to the LIF simulations, although here 50% of connections were randomly selected as inhibitory, meaning a population may send both inhibitory and excitatory inter-population connections. This level of inhibition was selected to prioritise network stability across a large range of random structures. A total of 100 structures were simulated. Excitatory connections resulted in the excitatory unit of the parent population influencing the excitatory unit of the child population (Fig. 2). Inhibitory connections resulted in the excitatory unit of the parent influencing the inhibitory unit of the child population (Fig. 2). Simulations were run for 1000 s, with a time-step (*dt*) of 1 ms. Data was averaged into 10 ms bins to provide 100,000 data-points for the structure learning process.

#### Simulation metrics

$CV_{ISI}$ and $CV_{IPI}$ values were calculated as the ratio of the standard-deviation to the mean of the inter-spike and inter-peak intervals, with a value close to 1 indicating irregular firing pattens. A cross-correlation analysis was used to estimate the true lag between connected nodes in the simulated networks. In this analysis, only nodes which were directly connected in simulations were tested. The lag at which the correlation between the parent and child was maximised was recorded, and the distribution of these values across all connections and simulations were considered for each simulation type.

**Table 2:** Parameters utilised in Wilson-Cowan simulations

Parameter	Description	Value
$\tau _E$	Excitatory time constant	10 ms
$\tau _I$	Inhibitory time constant	20 ms
$w_{EE}$	Relative strength of endogenous E $\xrightarrow {}$ E coupling	6.4
$w_{IE}$	Relative strength of endogenous E $\xrightarrow {}$ I coupling	4.8
$w_{EI}$	Relative strength of endogenous I $\xrightarrow {}$ E coupling	6
$w_{II}$	Relative strength of endogenous I $\xrightarrow {}$ I coupling	1.2
*α *	Relative strength of exogenous E $\xrightarrow {}$ E coupling	1
*β *	Relative strength of exogenous E $\xrightarrow {}$ I coupling	2
$\theta _{Act}^E$	Threshold of excitatory activation function	2.8
$\theta _{Act}^I$	Threshold of inhibitory activation function	4
$\alpha _{Act}^E$	Gain of excitatory activation function	1.2
$\alpha _{Act}^I$	Gain of inhibitory activation function	1
$\theta _{OU}$	Rate of mean reversion in OU noise	0.05
$\sigma _{OU}$	Volatility of OU noise	0.1
$\mu _{OU}$	Mean of OU noise	0.5
*dt*	Simulation time step	1 ms

### Learning network structure

#### Dynamic Bayesian networks

Dynamic Bayesian networks (DBNs) extend the principle of BNs across the temporal domain. In a DBN a node at a given time-point is dependent on itself at each previous time-point from the minimum to the maximum Markov lag, as well as parent nodes at any of these time points (Smith, 2010). The graphical representation of these nodes across time can then be reduced down to a single network, representing the cross-node dependencies in the dynamic network. The resultant network is able to overcome the key limitations of a static BN. Firstly, this network may contain cycles, as arcs may represent reciprocal relationships between nodes at different time points. Additionally, it is possible to confidently infer the direction of an arc, as causal influence must be exerted forwards in time. The maximum and minimum Markov lag in all discussed analyses is 1. To force the necessary connections within the DBN structure, forward links from a variable to the lagged version of itself are required to be present. This means that self-connections are always present and therefore are not considered in performance evaluation. Links backwards in time are prevented, asserting the assumption that influence moves forward in time. DBN structures were handled using the DBayesNet package, developed alongside this work to allow DBN structure learning in R with bnlearn (Scutari, 2010) and available at https://github.com/jacobhegarty/DBayesNet.

DBN structures may be learned in the same manner as static BNs. There are a range of score-based metrics available to evaluate network structures, a variety of which were evaluated here. These scores all evaluate the network structure as a whole for its ability to describe the data through calculating the probability of the candidate graphical structure given the observed data. Using Bayes theorem, with *G* representing the candidate graphical structure and *D* the observed data, this can be calculated as:

9 $$\begin{aligned} P(G|D) = \dfrac{P(D|G)P(G)}{P(D)} \end{aligned}$$

For the purposes of structure learning, the probability of observing the data *P*(*D*) is constant and thus ignored. In practice, the probability of the candidate graphical structure *P*(*G*) is often kept constant, although it can be utilised to include prior information over the likelihood of different graph structures. This leaves the probability of observing the data given it was generated by the candidate graphical structure *P*(*D*|*G*) to be calculated. This can be done using scores based on maximum or marginal likelihood, many of which have formulations for both continuous and discrete data. For discrete scores, quantile discretisation was performed to label data into three classes using the Hmisc library in R (version 5.2.3) (Harrell, 2025). All scoring metrics were applied using bnlearn (Version 5.0.2) (Scutari, 2010). See Section 2.3 for a brief overview of the scoring metrics tested here.

The number of possible network structures increases super-exponentially with the number of network nodes, and finding the best scoring network is an NP-hard problem (Chickering, 1996). This means that scoring of all networks to find the optimal solution is not possible, and heuristic search strategies must be utilised. Here, searches were performed in bnlearn using a hill-climb algorithm (Scutari, 2010), a method which has been widely employed in the literature (McNally et al., 2017; Owens et al., 2022; Tsukahara et al., 2022; Smith et al., 2006). This strategy is a type of greedy search which starts with a random network structure and randomly adds, deletes, or reverses the direction of a link. If this action improves the network score, it is accepted into the network structure. This process is repeated until no improvements to the network score can be made in this manner (Smith, 2010). This solution is not guaranteed to represent the overall best scoring network, as the search may have become stuck on a local minima, where network score cannot be improved by making a single change but does not represent the best network overall. In order to negate this risk, the hill-climb search was repeated 100 times, each beginning with a different random structure. Initial investigations indicated that this number of repeats was sufficient to find the same scores as longer searches, indicating that the best scoring network had been found.

#### Multivariate Granger causality

Conceptually, GC tests whether information on the past of variable *X* improves prediction on the future of variable *Y* beyond the past of *Y* itself (Granger, 1969; Geweke, 1982). In practice, this involves fitting a ‘reduced’ and ‘full’ model, the former being an autoregressive model for the dynamics of *Y* up to a maximum lag *L* and the latter a vector autoregressive (VAR) model of *Y* as a combination of the past of *Y* and *X* up to lag *L*. The residuals of these models can then be compared to calculate the Granger causality from *X* to *Y*. In the multivariate case (MVGC), the GC of *X* on *Y* is conditioned on other variables in the system, with these additional variables being included in both the full and reduced models (Geweke, 1984). Granger causality is computed as

10 $$\begin{aligned} GC_{Y \rightarrow X} = \text {max}\left( \ln \dfrac{\sum ^N_{t=L}\tilde{\epsilon }_t^2/(T_{regr} - M_r)}{\sum ^N_{t=L}\epsilon _t^2/(T_{regr} - M_f)},0\right) , \end{aligned}$$

where *N* is the number of time-points, *L* is the model order or lag, and $\tilde{\epsilon }_t$ and $\epsilon _t$ are the residuals from the reduced and full models at time t. This means that MVGC is calculated as the logged ratio of the sum of squared residuals from the reduced ($\tilde{\epsilon }$) and full (*ε *) models, normalized by the difference between the number of time points used in the models ($T_{regr}=T-L$) and the number of parameters in the corresponding reduced and full regression models $M_r$ and $M_f$ (where $M_r = M_f - L$).

Selection of model order *L* is important to balance the fit of the VAR model. Here, the elbow point of the average Akaike Information Criterion (AIC) value for all models in the network as a function of lag was used. Given that the underlying dynamics do not change between simulations, and computational cost of testing multiple lagged regressions for each potential connection across all datasets, this sub-analysis was limited to 10 simulations, with the value of *L* used on the whole dataset selected as the point at which increasing the *L* produced negligible change in average AIC for all simulations tested.

The significance of MVGC values can be utilised via comparing residuals $\tilde{\epsilon }_t$ and $\epsilon _t$ using an F-test. Two types of Granger causality networks were formed, “significant-only” with links selected based on significant GC values, and “non-zero” with links selected for all non-zero MVGC values. A 0.05 significance threshold was adjusted for the 90 potential connections within the network using a Bonferroni correction, meaning the criteria for a link in the significant-only MVGC networks was $p<\frac{0.05}{90}$.

#### LASSO regression

A LASSO regularisation forces the absolute values of regression coefficients to be less than the parameter *λ *, shrinking the coefficients of less important variables towards zero. In order to learn network structure, each node was modelled using all other neurons, with all nodes with non-zero coefficients selected as its parents. Optimal *λ * values were selected for each regression as the largest value which gives a model error within 1 standard error of the minimum. This method selects the simplest (least parametrised) model with accuracy within a reasonable distance of the most accurate model, thereby balancing avoidance of over-fitting and model accuracy (Friedman et al., 2010). LASSO regressions and *λ * selection were performed using the glmnet package in R (Version 4.1.8) (Friedman et al., 2010).

### Bayesian network scoring metrics

#### Maximum likelihood scores

A number of scoring metrics are based on estimating the probability of the data given the graph and the maximum likelihood estimation of the parameters ($\hat{\theta }$) from the data ($P(D|G,\hat{\theta })$).

##### Log-likelihood

The simplest of these is the log-likelihood, the logged value of the maximum likelihood (Koller & Friedman, 2009). For discrete data this is calculated as:

11 $$\begin{aligned} \ln (\hat{L})&= \ln (P(D|G))\\&=\displaystyle \sum ^n_{i=1}\sum ^{q_i}_{j=1}\sum ^{r_i}_{k=1}N_{ijk}\ln (P(X_i = k|\text {Pa}(X_i) = j)\nonumber \\ &= \sum ^n_{i=1}\sum ^{q_i}_{j=1}\sum ^{r_i}_{k=1} N_{ijk} \ln \frac{N_{ijk}}{N_{ij}}\nonumber \end{aligned}$$

where *n* is the number of nodes, $q_i$ is the number of parent set configurations for node $X_i$, $r_i$ is the number of possible states of node $X_i$, $N_{ijk}$ is the number of instances where the parents of $X_i$ ($\text {Pa}(X_i)$) are in configuration *j* and $X_i$ is in state *k*, and $N_{ij}$ is the number of times a the parent set of $X_i$ are in state *j* ($N_{ij} =\displaystyle \sum _{k=1}^{r_i}N_{ijk}$). Therefore, the discrete log-likelihood is the sum of the logged probability of each child state given each parent state, weighted by the number of times this combination occurs in the data.

For continuous data, assuming a joint Gaussian distribution with independent identically distributed observations, the log-likelihood score may be calculated as

12a $$\begin{aligned} \ln (\hat{L})&= \ln (P(D|G))\\&=\displaystyle \sum _{i=1}^n\sum _{o=1}^N -\frac{1}{2} \ln (2 \pi ) - \ln (\sigma _i) - \frac{(x_{io} - \hat{x}_{io})^2}{2\sigma _i^2} \nonumber \end{aligned}$$

where $x_{io}$ is the observed value of child node $X_i$ at observation *o*, $\hat{x}_{io}$ is the fitted value of linear regression of the parent variables on the child variable *i* ($\hat{\mathbf {x_i}}$) at observation *o*.

12b $$\begin{aligned} \hat{{\mathbf {x}}}_{i}&= \tilde{{\mathbf {Y}}}_{i}(\tilde{{\mathbf {Y}}}_{i}^{\top }\tilde{{\mathbf {Y}}}_{i})^{-1}\tilde{{\mathbf {Y}}}_{i}{x}_{i} \end{aligned}$$

and $\sigma _i$ is the standard deviation of residuals of this regression. $\tilde{Y}_i$ is all observations of $Pa(X_i)$ and $x_i$ is all observations of $X_i$.

Intuitively, log-likelihood will find the structure in which the parents contain the maximum possible information on the child. As such, this score favours complex structures, often learning fully connected networks, and is included here as a reference point. The reliance on linear regression in the continuous log-likelihood score means that this score and its penalised versions (below) are only able to capture linear relationships between nodes.

##### Akaike information criterion (AIC)

The Akaike information criterion (Akaike, 1998):

13 $$\begin{aligned} \text {AIC} = 2k - 2\ln (\hat{L}) \end{aligned}$$

extends this score to include a penalty term proportional to the number of parameters in the model (*k*). This penalty attempts to limit model complexity, with structures containing more connections being more strongly penalised.

##### Bayesian information criterion (BIC)

The Bayesian information criterion (Schwarz, 1978):

14 $$\begin{aligned} \text {BIC} = k\ln (N) - 2\ln (\hat{L}) \end{aligned}$$

penalises $\ln (\hat{L})$ in proportion to both *k* and the number of data points used in structure learning (*N*). The penalty term in BIC is more severe than that of AIC when *N>7*, which is generally the case in BN structure learning.

##### Extended Bayesian information criterion (eBIC)

The extended Bayesian information criterion (Foygel & Drton, 2010):

15 $$\begin{aligned} \text {eBIC} = k\ln (N) -2\ln (\hat{L})+4k\gamma \ln (n) \end{aligned}$$

score adds an additional penalty relative to both *k* and the size of the network (*n*). The parameter *γ * controls the severity of this penalty, and the default value of 0.5 was used here. In the structure learning process, the size of the network remains equal so this the additional penalty has the effect of scaling complexity penalisation by network size. For *γ = 0.5* and a network of 10 nodes, eBIC applies a more severe complexity penalty of *2łn (10)k* relative to BIC.

#### Marginal likelihood scores

As opposed to the aforementioned maximum likelihood scores, the Bayesian Dirichlet equivalent (BDe) and Bayesian Gaussian equivalent (BGe) scores do not rely on maximum likelihood estimation of parameters, or contain an explicit penalty term for complexity. They instead calculate the marginal likelihood of the data given the candidate graphical structure (*P*(*D*|*G*)) by integrating over the likelihood of the data given all possible values for parameters *θ * for this structure, weighted by a prior over the value of these parameters:

16 $$\begin{aligned} P(D|G) = \int \limits _{\theta } P(D|G, \theta ) P(\theta |G)d\theta \text {.} \end{aligned}$$

Bayesian Dirichlet (BD) scores (Heckerman et al., 1995) are utilised for discrete data, and contain a Dirichlet prior (the conjugate prior of the multinomial distribution) for the distribution of probabilities for each state occurring in a node given its parents’ states, for all possible combinations and configurations of parents. This results in a network being scored as:

17 $$\begin{aligned} \begin{aligned} BDe(G) = \log \Bigg {(}&\prod ^n_{i=1}\prod ^{q_i}_{j=1}\dfrac{\Gamma (\alpha _{ij})}{\Gamma (\alpha _{ij} +N_{ij})} \; \times \\&\prod _{k=1}^{r_i}\dfrac{\Gamma (\alpha _{ijk}+N_{ijk})}{\Gamma (\alpha _{ijk})}\Bigg {)}\text{. } \end{aligned} \end{aligned}$$

Where *n* represents the number of nodes in the network, $q_i$ the number of parent configurations for node $X_i$, and $r_i$ the number of states of node $X_i$. The hyper-parameter ${\alpha _{ijk}}$ controls the Dirichlet distribution, and $\alpha _{ij} = \sum _{k=1}^{r_i}\alpha _{ijk}$. As in the discrete log-likelihood score, $N_{ijk}$ is the number of times each child-parent configuration combination is observed in the data, $N_{ij} = \sum _{k = 1}^{r_i}N_{ijk}$. Intuitively, the second product is higher when the parents of each node best predict the corresponding child, and the first product is higher when there are fewer parents for each node and with an equal number of observations of each parent configuration. The first term, therefore, provides an implicit penalty for the complexity of the structure. For BDe the parameters of the Dirichlet prior are calculated from the the imaginary sample size (iss) parameter *α *, which was set to 1 for all analysis in this study, via the equation: $\alpha _{ijk} = \dfrac{\alpha }{r_iq_i}$. Utilising a uniform prior removes the need to subjectively specify a prior for each nodes’ potential parent configurations and assumes that all BN structures are equally likely. In reference to this, BDe may also be referred to as BDeu in the literature, throughout this paper we refer to BDeu as BDe. It is worth noting that BDe is closely related to another popular BN scoring algorithm K2, which uses $\alpha _{ijk} = 1$ (Cooper & Herskovits, 1991).

The BGe score (Geiger & Heckerman, 1994) is used for continuous data, utilising a Normal-Wishart distribution (the conjugate prior of a multivariate Gaussian distribution) for the mean and precision matrix parameters of the joint normal distribution over each parent-child node set. This results in a network being scored as:

18a $$\begin{aligned} BGe(G)&= \log \Bigg {(}\displaystyle \prod _{i=1}^n \left( \dfrac{\alpha _\mu }{N+\alpha _\mu } \right) ^{\frac{1}{2}} \; \times \nonumber \\&\qquad \qquad \qquad \,\,\,\dfrac{\Gamma \big (\frac{N +\alpha _w - n +p_i+1}{2}\big )}{\pi ^{\frac{N}{2}}\Gamma \big (\frac{\alpha _w - n +p_i+1}{2}\big )} \; \times \nonumber \\&\qquad \qquad \qquad \,\,\, t^{\frac{\alpha _w - n +2p_i+1}{2}} \;\times \nonumber \\&\qquad \qquad \qquad \,\,\,\dfrac{|R_{Y_iY_i}| ^{\frac{N +\alpha _w - n +p_i}{2}}}{|R_{\tilde{Y_i}\tilde{Y_i}}{|^\frac{N+\alpha _w-n+p_i+1}{2}}} \Bigg {)}, \end{aligned}$$

where

18b $$\begin{aligned} \qquad \qquad t = \dfrac{\alpha _\mu - n - 1}{\alpha _\mu +1}, \end{aligned}$$

$R_{Y_iY_i}$ is the posterior scale matrix of parent and child nodes,

18c $$\begin{aligned} R_{Y_iY_i}&= \displaystyle \sum _ {o=1} ^N (\boldsymbol{x_o}^{Y_i} - \boldsymbol{\bar{x}}^{Y_i} )(\boldsymbol{x_o}^{Y_i} - \boldsymbol{\bar{x}}^{Y_i} )^\text{ T } \; + \nonumber \\&\quad tI_n \; +\nonumber \\&\quad \, \frac{N\alpha _\mu }{N + \alpha _\mu }(\boldsymbol{\nu }^{Y_i}-\boldsymbol{\bar{x}}^{Y_i} )(\boldsymbol{\nu }^{Y_i}-\boldsymbol{\bar{x}}^{Y_i})^\text{ T } \end{aligned}$$

and $R_{\tilde{Y}\tilde{Y}}$ is the same posterior scale matrix without the child node $X_i$:

18d $$\begin{aligned} R_{Y_iY_i}&= \begin{pmatrix} R_{X_iX_i} & R_{X_i\tilde{Y_i}}\\ R_{\tilde{Y}Xi} & R_{\tilde{Y_i}\tilde{Y_i}}\end{pmatrix}. \end{aligned}$$

As in BDe, *N* is the total number of observations, and *n* the total number of nodes, in the data. $p_i$ is the number of parents of node $X_i$ in the candidate structure. Set $Y_i$ contains the node $X_i$ and its parents in the candidate structure, and $\tilde{Y_i}$ contains only the parents of node $X_i$. $\boldsymbol{x}_o^{Y_i}$ is the vector of observations of all nodes in $Y_i$ at observation *o*. $\boldsymbol{\bar{x}^{Y_i}}$ is a vector containing the mean values of nodes in $Y_i$, and $\boldsymbol{\nu }^{Y_i}$ is the vector containing the priors over node means for nodes in Y. In this work, the prior over node means is calculated as the mean value of that node, meaning the last term in the calculation of $R_{Y_iY_i}$ disappears. $R_{Y_iY_i}$ is therefore the un-normalised covariance, or scatter matrix, of $Y_i$, with *t* added to the diagonal values of this matrix, corresponding to the variance of each variable in $Y_i$, via addition of the product of *t* and an identity matrix of size n ($I_n$). $R_{\tilde{Y}_i\tilde{Y}_i}$ is calculated in the same manner, but for set $\tilde{Y}_i$. The final term in the BGe calculation therefore measures how much adding the child to its parents increases the total covariance. If the child is well explained by its parents, this increase is small, and the score is higher. The previous terms come from integration over parameters and the Normal-Wishart prior, and include an implicit penalty for structure complexity and lack of observations. $\alpha _{w}$ and $\alpha _{\mu }$ are the equivalent sample size hyper-parameters controlling this prior over the spread of the precision matrix and mean respectively. These were set to *n+2* and 1 for all work presented here. As with the Gaussian log-likelihood based scores, data are assumed to be sampled from a joint Gaussian distribution, with independent and identically distributed observations. This results in the score only accounting for linear relationships between variables.

### Performance evaluation

Performance was evaluated using precision and recall. Precision measures the ability of a method to find true links without finding false links (19).

19 $$\begin{aligned} \text {Precision} = \dfrac{\text {True Positives}}{\text {True Positives}+{\text {False Positives}}} \end{aligned}$$

True positives are correctly identified links and false positives are links which the structure learning method predicted to exist but were not present in the ground-truth network. Recall measures the ability of the method to find true links (20). High precision and low recall indicate under-fitting, whereas low precision and high recall indicate over-fitting.

20 $$\begin{aligned} \text {Recall} = \dfrac{\text {True Positives}}{\text {True Positives}+{\text {False Negatives}}} \end{aligned}$$

False negatives are cases where the structure learning method failed to identify the presence of a specific link in the ground-truth network. It is important to consider both of these statistics together as a high score in one but not the other cannot indicate high performance. High recall with low precision is characteristic of over-fitting, and low recall with high-precision is characteristic of under-fitting in network structures.

Hypergeometric testing was also performed to ascertain the probability of achieving the same or more true positives as a method, given the number of arcs predicted in the learned network, if picking at random. This probability can be calculated directly from the cumulative density function of the hypergeometric distribution as:

21 $$\begin{aligned} P(X\ge k) = 1 - \displaystyle \sum _{i=0}^{k-1} \dfrac{\left( {\begin{array}{c}K\\ i\end{array}}\right) \left( {\begin{array}{c}N - K\\ n-i\end{array}}\right) }{\left( {\begin{array}{c}N\\ n\end{array}}\right) } \end{aligned}$$

Here *K* is the total number of connections present in the underlying ground-truth network, *N* is the number of potential connections in the network, *n* is the number of connections predicted by the structure learning method and *k* is the number of true positives. This test was performed separately for each score on each simulation, providing a distribution of performance, whereby a low probability indicates the method performed better than chance.

## Results

### Single-neuron data

**Fig. 3:**
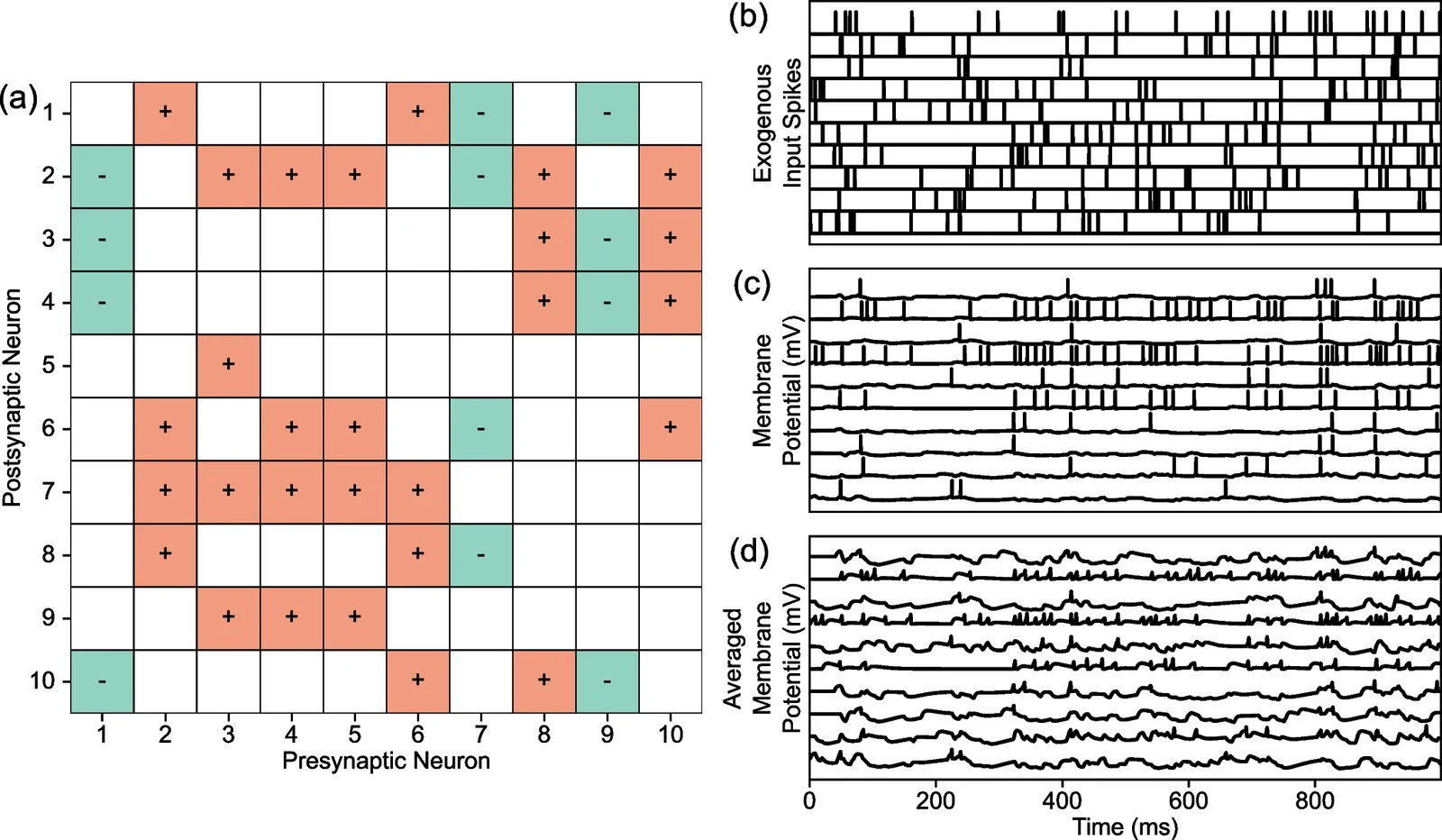
Example figures from a single leaky integrate and fire simulation run. (**a**) Example leaky integrate and fire network structure. Red squares indicate excitatory connections and green squares indicate inhibitory connections. (**b**) Example exogenous input to leaky integrate and fire networks. (**c**) Example simulated membrane potential trace made using network structure and input shown in a and b. (**d**) Simulation in c averaged in 1 ms bins, as used in structure learning process

Figure 3 shows a one second example LIF simulation. The mean $CV_{ISI}$ of LIF simulations was 0.93 (± 0.19 across simulations), with an average within simulation standard deviation of 0.20 (± 0.11 across simulations). The mean firing rate was 35.77 Hz (± 21.51 Hz across simulations), with a mean within simulation variation of 26.32 Hz (± 14.67 Hz across simulations). This indicates that the simulated structures produced a large range of network dynamics. A cross-correlation analysis found a median and mode optimal lag of 0 between all connections present in every simulation.

**Fig. 4:**
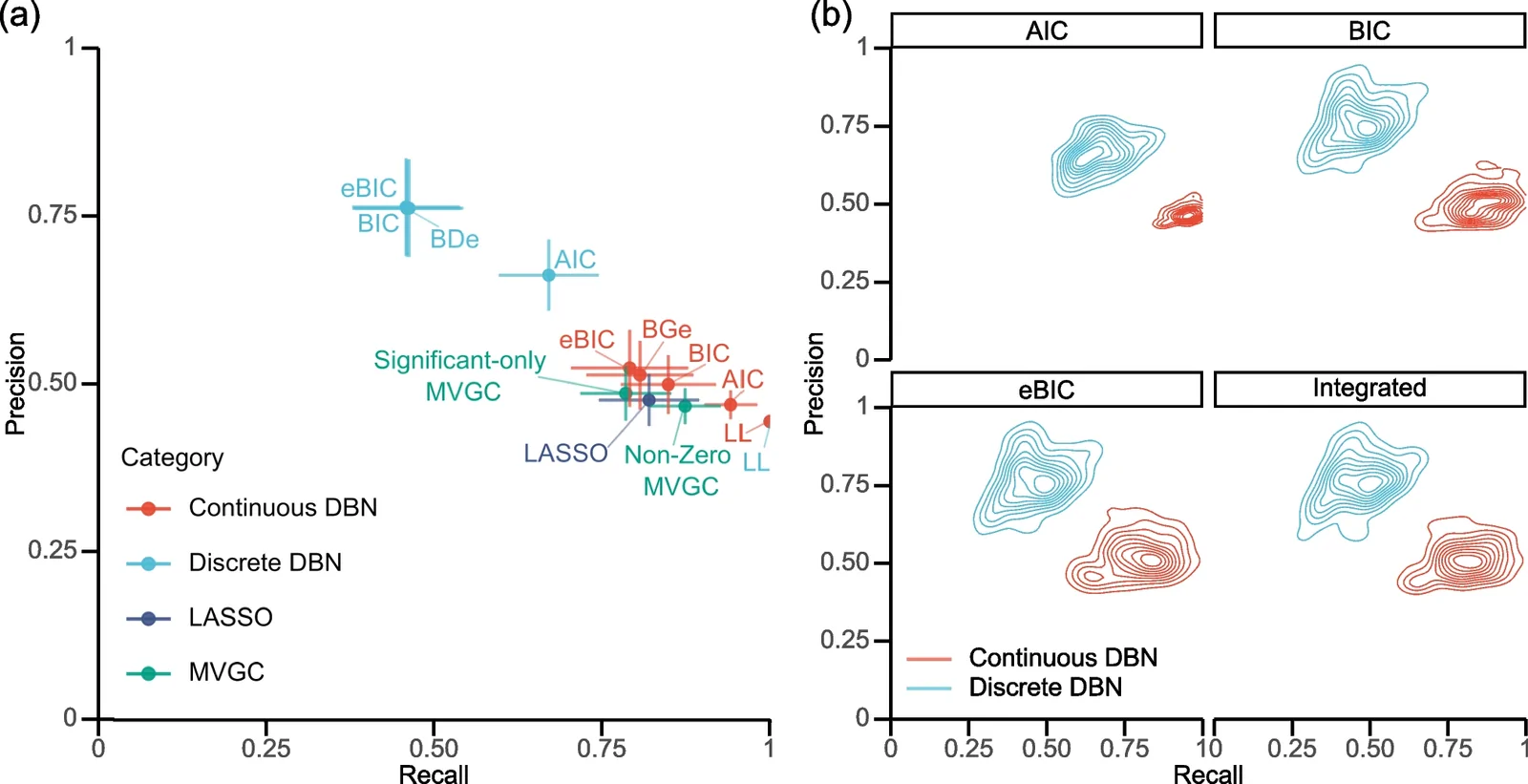
**a**) The average performance of discrete (blue) and continuous (red) DBN scores (eBIC = extended Bayesian information criterion, BIC = Bayesian information criterion, AIC = Akaike information criterion, LL = log-likelihood, BDe = Bayesian Dirichlet equivalent, BGe = Bayesian Gaussian equivalent), LASSO (purple) and MVGC (green) across 100 single-neuron leaky integrate and fire simulations when compared to ground truth connectivity, plotted in precision-recall space with recall on the x-axis and precision on the y-axis. **b**) The distribution in performance of DBN score types on discrete and continuous data for 100 single-neuron simulations, when compared to ground truth connectivity. Produced using a 2-D kernel density estimate calculated independently for each group with 10 bins separated by contour lines representing increasing density

The average performance of all tested structure learning methods on the single-neuron data in precision-recall space is shown in Fig. 4. Both log-likelihood scores completely over-fit these data, with high recall (both 1.00) and low precision (both 0.44) (Fig. 4). This is due to them learning fully connected network structures 100% of the time with discrete data, and 93% of the time with continuous data.

**Fig. 5:**
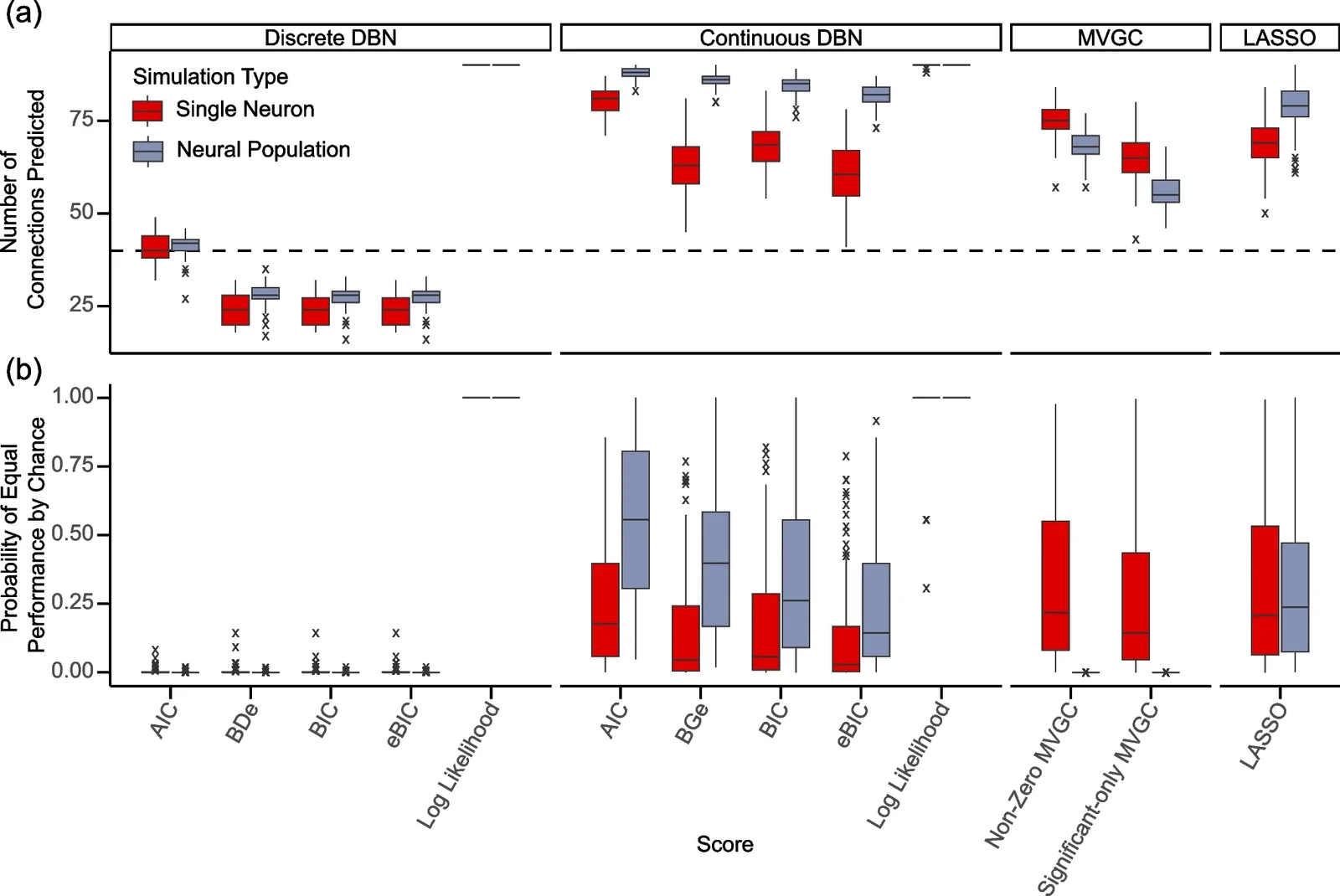
**a**) Number of connections predicted to be in structure underlying simulated single-neuron (red, N=100) and neural-population data (purple, N=100). Dashed line indicates the true number of connections in each network (40). **b**) Probability of predicting the same or more correct connections as each structure learning method, given the number of connections they predicted, from simulated single-neuron (red, N=100) and neural-population data (purple, N=100). Calculated using the hypergeometric distribution. Central hinge represents median value. Lower and upper hinges correspond to first and third quartiles. Whiskers indicate the furthest point within a 1.5 interquartile range from the hinge. Crosses indicate outliers

Mean precision and recall for discrete BIC, eBIC and BDe cluster closely in both precision and recall, all with an average precision of 0.76 and recall of 0.46 (Fig. 4). This indicates an under-fit of this dataset. Discrete AIC achieves a more balanced performance, with an decreased average precision (0.66) and increased average recall (0.67) (Fig. 4). Interestingly, discrete AIC predicts closest to the true number of connections in the network, with an average of 40.60, compared to the ground truth of 40. All other discrete scores, other than log-likelihood, underestimate the number of connections present (Fig. 5). Hypergeometric testing indicated a low average probability of achieving equal or better performance by chance for all discrete scores other than log-likelihood (all mean p<0.01; Fig. 5).

Continuous DBNs sit much further to the right in precision-recall space, with higher recall and lower precision. There is more separation in the performance of these score’s than their discrete counterparts, with increasing recall and decreasing precision from eBIC (precision = 0.52, recall = 0.79), to BGe (precision = 0.51, recall = 0.81), to BIC (precision = 0.50, recall = 0.85), to AIC (precision = 0.47, recall = 0.94) (Fig. 4). The continuous scores tended to overestimate the number of connections in the network (Fig. 5). Hypergeometric testing indicated a moderate probability of achieving equal or better performance by chance for the continuous scores other than log-likelihood, with AIC having the highest probability at 0.26 with a large standard deviation of 0.25, and eBIC the lowest at 0.13 with a standard deviation of 0.20. (Fig. 5). Aside from log-likelihood, there is a clear separation in precision-recall space between the continuous and discrete version of each score with continuous scores over-fitting and discrete scores under-fitting data (Fig. 4).

Optimal model order for MVGC was estimated as 10 (Fig. 8). Average recall for significant-only MVGC networks was 0.79, with an average precision of 0.49. For non-zero GC networks, average recall increased to 0.87 and average precision decreased to 0.47. Viewed in precision-recall space, it is clear that these methods perform extremely poorly on the single-neuron simulations (Fig. 4). Both significant-only and non-zero MVGC tended to over-estimate network density, with 64.87 and 74.91 connections respectively (Fig. 5). Hypergeometric testing further indicates high probabilities of achieving these levels of performance by chance, with an average of 0.33 for non-zero and 0.25 for significant-only networks. (Fig. 5).

The non-lagged LASSO method had an average recall of 0.82 and precision of 0.48 on the LIF dataset (Fig. 4). This method tended to over-estimate the number of connections, with an average of 69.07 (Fig. 5), indicating a clear over-fit of this dataset. The average probability of observing the same performance by chance was 0.31 (Fig. 5).

### Neural-population data

**Fig. 6:**
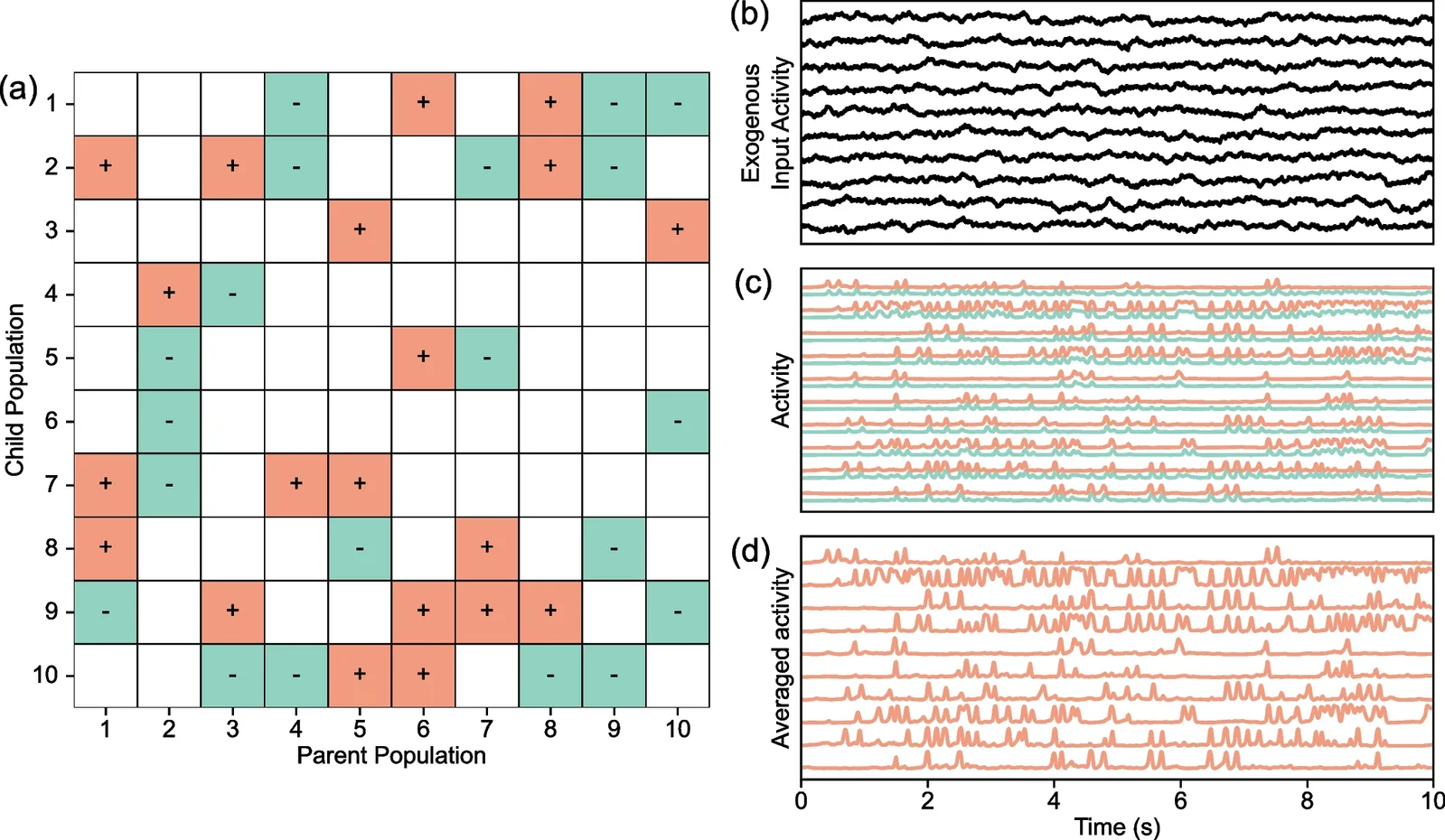
Example figures from a single Wilson-Cowan simulation run. (**a**) Example Wilson-Cowan network structure. Red squares indicate excitatory connections and green squares indicate inhibitory connections. (**b**) Example exogenous input to Wilson-Cowan network. (**c**) Example simulated population activity trace made using network structure and input shown in a and b. Excitatory unit activity shown in green, and inhibitory unit activity shown in red. Excitatory activity is offset vertically to aid visualisation. **d**) Excitatory trace averaged in 10 ms bins, as used in structure learning process

Figure 6 shows a 10 second example WC simulation. Simulations displayed patterns of chaotic activity. A cross-correlation analysis comparing connected excitatory populations in all networks gave a distribution of optimal lags with a median of 1 and a mode of 2.

The mean $CV_{IPI}$ of excitatory neurons in neural-population simulations was 0.60 (± 0.08 across simulations), with an average within simulation standard deviation of 0.17 (± 0.04 across simulations). The mean number of peaks in per second was 4.68 (± 0.533 across simulations) excitatory traces, with a mean within simulation variation of 1.38 (± 0.443 across simulations). The mean $CV_{IPI}$ of inhibitory neurons in neural-population simulations was 0.55 (± 0.09 across simulations), with an average within simulation standard deviation of 0.16 (± 0.07 across simulations). The mean number of peaks per second in inhibitory subpopulations was 5.11 (± 0.557 across simulations) in inhibitory traces, with a mean within simulation variation of 1.08 (± 0.446 across simulations).

**Fig. 7:**
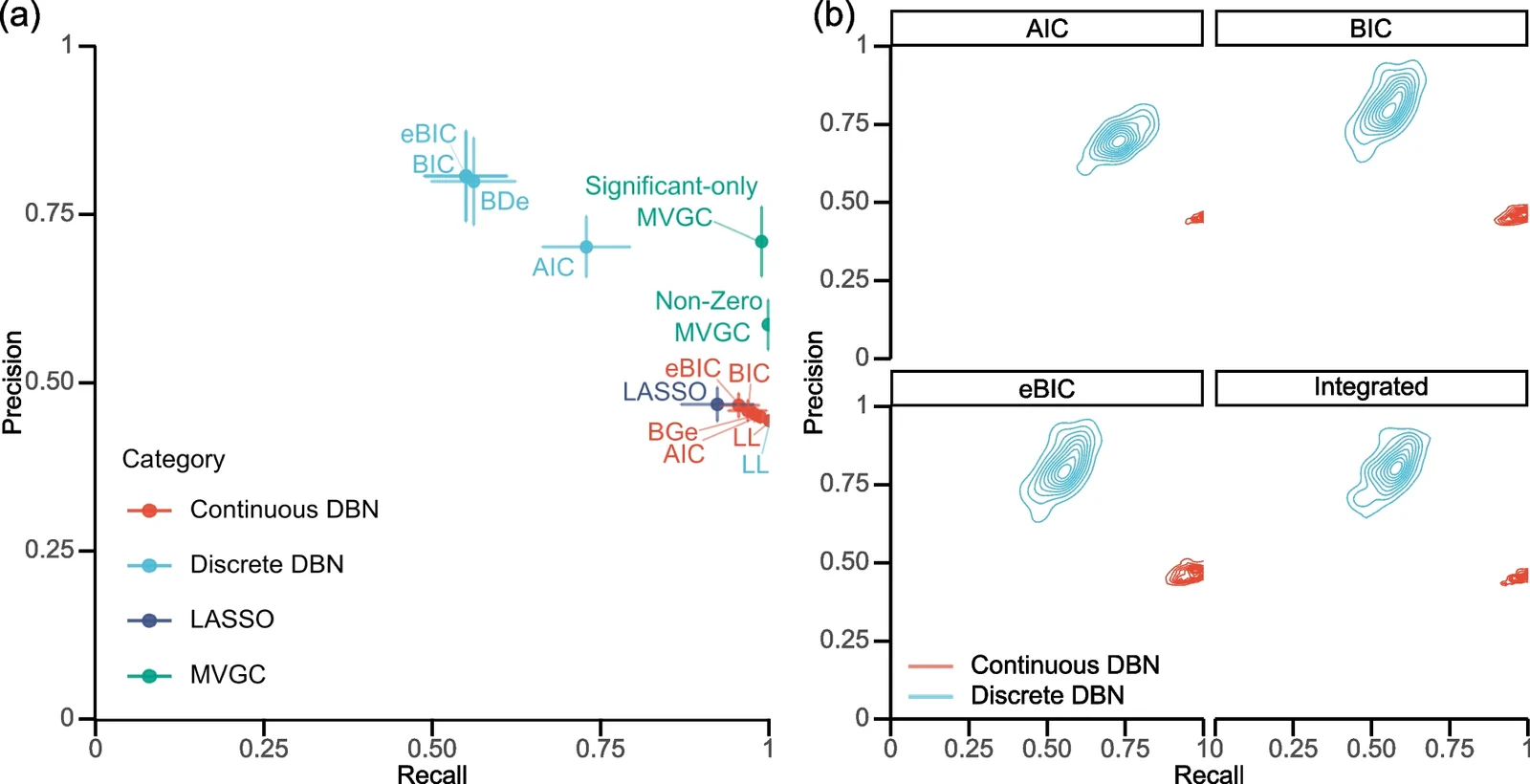
**a**) The average performance of discrete (blue) and continuous (red) DBN scores (eBIC = extended Bayesian information criterion, BIC = Bayesian information criterion, AIC = Akaike information criterion, LL = log-likelihood, BDe = Bayesian Dirichlet equivalent, BGe = Bayesian Gaussian equivalent), LASSO (purple) and MVGC (green) across 100 neural-population Wilson-Cowan simulations, when compared to ground truth connectivity. **b**) The distribution in performance of DBN score types on discrete and continuous data for 100 neural-population simulations, when compared to ground truth connectivity. Produced using a 2-D kernel density estimate calculated independently for each group with 10 bins separated by contour lines representing increasing density

The average performance of all tested structure learning methods on the neural-population data is shown in Fig. 7. As with the single-neuron data both the discrete and continuous log-likelihood scores over-fit data, both learning fully-connected network structures for every neural-population simulation (Fig. 5).

Discrete eBIC, BIC and BDe scores cluster closely in precision-recall space, eBIC and BIC with a recall of 0.55 and precision of 0.81 and BDe with a recall of 0.56 and precision of 0.80 (Fig. 7). As with the single-neuron data, discrete AIC was the best performing BN scoring metric for neural-population data, with an average precision of 0.70 and recall of 0.73 (Fig. 7). Discrete AIC predicted 41.50 connections on average, and all other discrete scores other than log-likelihood under-estimated network density (Fig. 5). Hypergeometric testing indicated that all discrete scores other than log-likelihood had a low average probability of being equalled or outperformed by chance (all mean p<0.001; Fig. 5).

Continuous scores all appear to over-fit neural-population data, with very high recall and low precision (AIC precision = 0.45, recall = 0.99; eBIC precision = 0.47, recall = 0.95; BIC precision = 0.46, recall = 0.97; BGe precision = 0.45, recall = 0.98) (Fig. 7). Continuous scores overestimated network density, all predicting over 80 connection on average (Fig. 5), with AIC learning fully connected structures 7% of time, BGe 1% and all other scores 0%. The average probability of observing the same performance as these methods by chance was 0.54 for AIC, 0.41 for BGe, 0.34 for BIC and 0.25 for eBIC (Fig. 5).

**Fig. 8:**
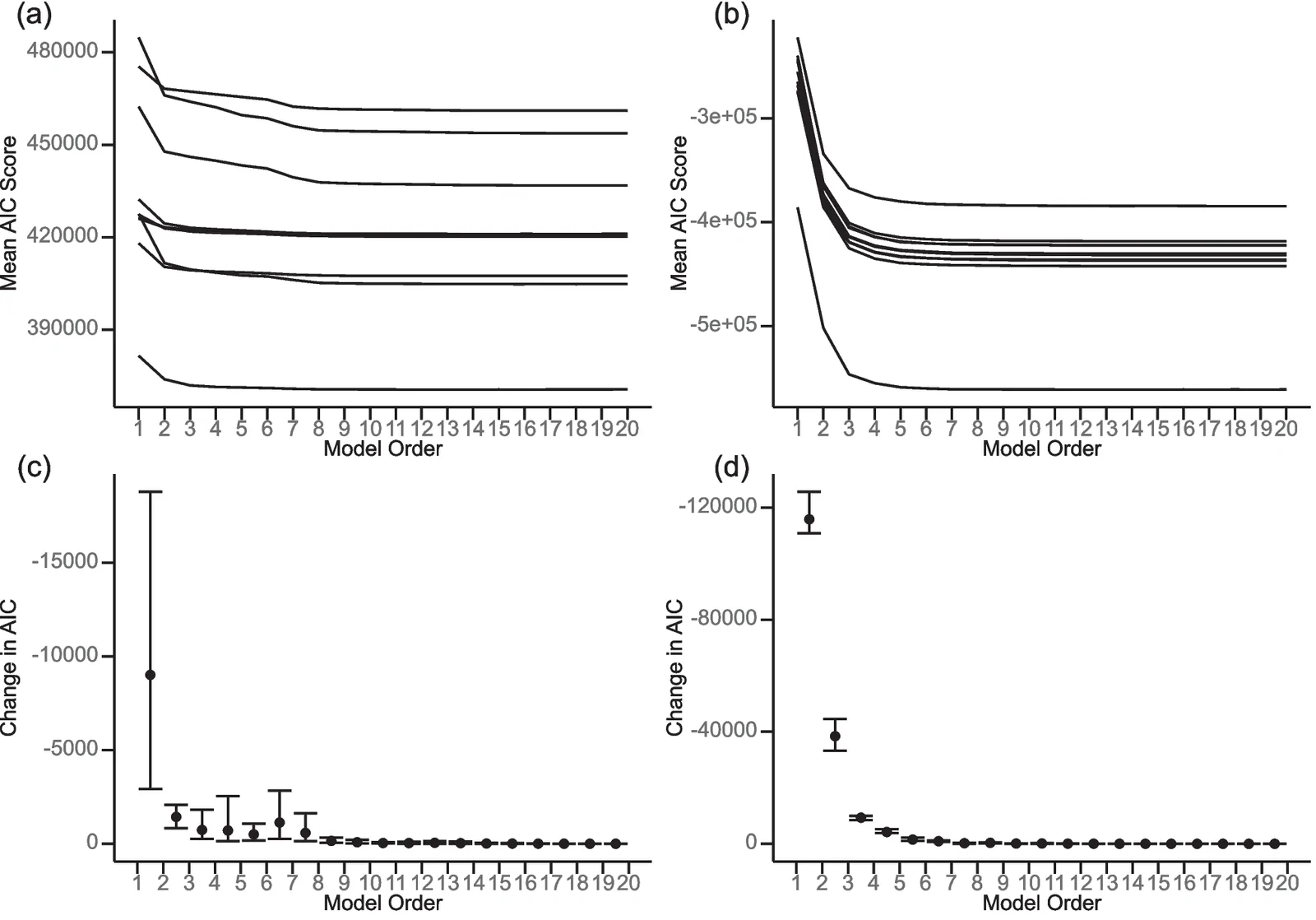
The upper panel shows average AIC values for full model of every node in 10 **a**) single-neuron and **b**) neural-population simulations with increasing model order, with each line representing a single simulation. The lower panel shows change in average AIC with increasing model order for the same 10 **c**) single-neuron and **d**) neural-population simulations, with error bars representing the range across the 10 simulations tested. Optimal model order for each data type was selected as the minimum value past which increasing order produces negligible change in AIC

Optimal model order for MVGC analysis was estimated as 7 (Fig. 8). Significant-only MVGC achieved an average precision of 0.71 and average recall of 0.99. Non-zero MVGC had a higher average recall of 1, but lower average precision of 0.59 (Fig. 7). Both significant-only and non-zero MVGC tended to over-predict network density, with an average of 55.98 and 68.33 predicted connections respectively (Fig. 5). Hypergeometric testing indicated an extremely low average probability of either of these methods being equalled or outperformed by chance (both mean p<0.0001; Fig. 5).

The non-lagged LASSO method had a mean recall of 0.92, and close-to-chance average precision of 0.47 (Fig. 7). This method tended to overestimate network density, with 78.96 connections predicted on average (Fig. 5) and 1% of learned networks fully connected. The average probability of achieving the same or better performance as LASSO by chance was 0.30 (Fig. 5).

## Discussion

We have evaluated the relative performance of discrete and continuous DBNs, MVGC and unlagged LASSO regressions in learning the structures underlying simulated single-neuron and neural-population data. We have found continuous DBNs to over-fit both data types, with a large number of false positive connections being learned, to the extent of performing close to chance on the neural-population data. Discrete DBNs achieve a more balanced performance on both datasets, although have a tendency to produce false negatives. MVGC performs poorly on single-neuron data, but well on neural-population data, especially when significance testing is utilised. Un-lagged LASSO regression performs extremely poorly, strongly over-fitting both of these data types.

### Precision-recall trade off is related to penalty for complexity in Bayesian network scores

Where they separate in performance, the penalised log-likelihood scores follow a clear pattern of increasing connection density of learned networks from eBIC to BIC to AIC. This reflects relaxation of the complexity penalty through this sequence of scores, from *7kłn (10)* in eBIC, to *5kłn (10)* in BIC, to 2*k* in AIC. Interestingly, the positioning of the integrated scores within this trend appears to be dependent on data type and vary between BDe and BGe. BDe appears to cluster closely with eBIC and BIC on both datasets, slightly outperforming these scores on the neural-population data. The similarity in performance of BIC and BDe, both learning sparser structures than AIC, aligns with previous findings (Ke et al., 2025). BGe sits between eBIC and BIC in precision-recall space on single-neuron data and over-fits slightly more than these scores in the population data. As expected due to their lack of penalty for complexity, the log-likelihood score tends to learn fully connected structures, and is not a useful metric for network structure learning in this context. Although discrete AIC appears to achieve the best balance of precision and recall of all BN scoring metrics on both data types, the most appropriate scoring metric for a given study will be highly contextual. Given the heavy trade-off between precision and recall for all metrics, particularly on the single-neuron data, the researcher must choose the acceptable balance in risk of missing true versus finding spurious connections.

### Discretisation reduces over-fitting with Bayesian network scores

There is a clear separation of discrete and continuous score performance across all BN scores tested and both data types. These results suggest that the increase in information contained in continuous activity traces lead to over-fitting of data. This over-fitting may be attributed to the small noise fluctuations in continuous traces which would not have affected discretised labels. We performed a sub-analysis, which found no change in the performance of continuous DBNs with increasing observation noise, likely due to continued over-fitting of data (SI 1). In the same analysis discrete DBNs were the most robust method overall to increasing observation noise in neural population data. This is in contrast to Wu et al. (2013), who found that Gaussian DBNs learned using the BIC score were more robust to increasing noise than their discrete counterparts. This difference in findings may be due to the increased complexity of simulations utilised here, with Wu et al. (2013) utilising linear multivariate autoregressive models which may have favoured Gaussian over discrete DBNs. In single-neuron data, continuous networks may offer an advantageous increase in recall for scoping studies where reliability of findings is less important than identifying potential connections. In population level data, however, the over-fitting effect is so extreme that results are unlikely to be useful in any context. This result is surprising given the increased coherence of the population model to the assumption of linearity of continuous DBNs.

### Data type influences relative performance of methods

The contrast in relative performance of DBNs and MVGC on different data types, with MVGC the best performing on more linear neuronal systems and DBNs on more non-linear systems, provides guidance on the most appropriate methods in these situations. The fact that the most common optimal lag for neural-population data based on cross-correlation analysis was greater than the maximum Markov lag used in DBN structure learning, but less than the model order used in MVGC, may explain the increase performance of the latter method on this data type. Unfortunately, learning DBN structures with a lag comparable to that used by MVGC is generally unfeasible. For example, a model order of 7 in MVGC corresponds to a DBN with a minimum Markov lag of 1 and a maximum Markov lag of 7. In a rolled network of 10 nodes, this corresponds to a dynamic network of 80 nodes and $2^{1890}$ potential structures, and the computational complexity of searching heuristically across a space this large would make structure learning unfeasible. On single-neuron data, however, DBNs outperform MVGC, illustrating differences in the ability of these methodologies to capture more noisy non-linear neuronal dynamics. This may have been expected due to the reliance of MVGC on linear modelling (Dang et al., 2015), however contrasts with the results of Chen et al. (2023) that MVGC may still be suitable for quantifying interactions in non-linear systems. The latter contrast may be due to an increased degree of non-linearity in the simulations, with Chen et al. (2023) using a calcium observation function which involves summation over a neurons spikes over time. Further, this study only tests a single network structure, meaning some of this disparity in findings may reflect the more diverse simulation dynamics tested here. Unsurprisingly, the un-lagged LASSO method performs poorly on both datasets, highlighting the importance utilising lag-based methods for NIF analysis on any data type.

In single-neuron data, where MVGC performance is poor, significance testing of links appears to cause a decrease in overall performance, with a moderate increase in average precision accompanied by a large decrease in recall. Clearly, in this case more true positives than false positives were filtered out by significance testing. In contrast, when viewed in precision-recall space it is clear that significant-only MVGC achieves the best performance on neural-population data. In this case non-zero MVGC performs with a much lower average precision, suggesting that here significance testing has successfully filtered out false positive links. The difference in effects of significance testing on MVGC performance between data types illustrates the difficulties in thresholding link selection in pairwise methods, and the ambiguity of proper statistical techniques in such conditions. This is further highlighted by our supplementary analysis into the effects of network size on structure learning performance, in which precision of non-zero MVGC decreases with increasing network size, whereas significant-only MVGC increases slightly (SI 2). This suggests that significance testing within MVGC is both more effective and effective in larger networks. This may be due to the Bonferroni correction applied during significance testing, which scales with network size and prompts further study into the effectiveness of this adjustment across network sizes.

### Generalisability of findings

A number of network and analysis level variables which may have affected the dynamics of the simulated data and performance of the structure learning methods were not varied in the main analysis. We investigated some of these in supplementary analyses (SI 1-5).

The amount of noise associated with the experimental method utilised in data collection at the level of observation may vary in real data. As previously mentioned, a sub-analysis was performed, adding increasing levels of white-noise to a small subset of simulations and retesting structure learning performance (SI 1). In single-neuron data, all methods reduced in precision with increasing observation noise, although their relative positioning in precision recall space remained constant. In neural-population data, MVGC methods suffer a large decrease in precision with increasing observation noise. Discrete DBNs remain relatively stable in performance, further promoting them as a robust tool for NIF analysis. NIF networks may differ in number of nodes based on the study species and spatial scale and resolution experimental methods utilised.

To test for consistency in relative structure learning performance at different network sizes, a supplementary analysis was performed on a small number of 20 and 50 node networks (SI 2). This analysis showed discrete DBNs to maintain precision with increasing network size for single-neuron simulations, where all other methods reduced in line with chance. In neural population data, both discrete DBNs and significant-only MVGC are robust to this effect. These findings further promote discrete DBNs as a robust methods for NIF structure learning across network sizes, particularly with single-neuron data.

Density of connections varies between neuronal circuits and has an effect on activity patterns (Ivenshitz & Segal, 2010). Given the patterns of over- and under-fitting observed by various score categories, this density may have an effect on score performance. An additional analysis was performed on a smaller number of simulations to test this, finding consistency in the relative positioning of methods in precision recall space at various network densities (SI 3).

In NIF networks, levels of inhibition are likely to vary (Douglas & Martin, 2009; Tatti et al., 2017), and this may effect the performance of the structure learning methods tested here. A scoping sensitivity analysis was performed to test this, and found the relative positioning of methods in precision recall space to be stable (SI 4).

Availability of data may also vary based on experimental method utilised, and could have a large impact on structure learning performance (Zuk et al., 2006). To test the consistency of relative score performance in precision recall space, a sub-analysis was performed on a small number of simulations with a reduced number of data-points. Variance in performance increased with decreased data availability in both data types. In neural population data, discrete BIC and eBIC fail to identify any connections. There also appears to be a movement from under-fitting towards over-fitting with increasing data availability in discrete DBNs and MVGC, most severely effecting MVGC. This prompts future study on how amount of data effects structure learning performance, and potential strategies for limiting the over-fitting associated with increased data (SI 5).

This study has provided an initial investigation of DBN structure learning performance on neuronal data. These models are meant to be generally biologically plausible, but are not intended to emulate any specific neural network. Moreover, the random structures produced a wide range of network dynamics. This is evident from the large between-sample variance in mean spiking rate and frequency indicate a range of excitability in network structures. Further, large between-sample variance in the CV for spikes and peaks indicates a range in regularity of activity across the datasets. This variance improves the generalisability of our findings to applications across nervous systems, and was the main justification for utilising random, as opposed to more stable or predefined biological network structures. However, this means that structures do not correspond to a specific biological network. The parameters utilised within simulations were selected to produce stable, irregular dynamics across a range of random structures. This removed biological relevance from some of the parameters utilised, as the processes they represent are likely to vary between circuits *in vivo*. Additionally, the variation in network structure may have produced subtle differences in the lag of information flow. This means that estimated optimal model order in MVGC may differ slightly between simulations, and use of the same model order within simulation types may have effected these results. However this effect will be minimal, given that parameters remain constant through simulations.

### Practical recommendation based on research goals

Given the evidence collected here, the choice of the best structure learning method depends on the researcher’s goals and data. Spiking neuronal data is highly noisy and non-linear, and DBNs appear to deal with these data better than MVGC. For a scoping study, aiming to identify potential connections within a network where precision is not important, continuous DBNs may be preferable. For studies which require increased confidence in the validity of learned structures, discrete DBNs will be more suitable. The discrete AIC achieves the most balanced performance. As more neurons’ spiking activity is collated, for example by methods from calcium imaging to EEG, and data becomes increasingly linear (Deco et al., 2008), MVGC with significance testing achieves the best performance. Methods focusing on association between variables are unsuitable for NIF network structure learning due to an inability to infer directionality of influence (Camps-Valls et al., 2023). This is demonstrated by the poor performance of the un-lagged LASSO regression based method presented here.

### Directions for future work

In real neuronal data, circuits at the single-neuron level are continuously remodelled by plasticity, which may affect NIF network structure or parametrisation (Abbott & Regehr, 2004). In optimal conditions, the learned DBN will represent the strongest pathways of information flow present across the data available. Small changes in the strength of synaptic connections would therefore be expected to have little impact on overall NIF network structure and would be averaged out into the learned DBN (Burge et al., 2007). These changes may impact the stationarity of data, and the effects of the violation of this DBN assumption remain unknown. Longer-term changes in synaptic connectivity or strength may represent a shift in the structure of the NIF network, and in these cases it will be advantageous to learn distinct NIF structures representing the different NIF states. Non-stationary DBNs represent a promising area for such analysis, and further work may focus on applying this methodology in these situations.

The dynamics underlying the single-neuron data were much quicker than the data resolution utilised in structure learning, with cross-correlation analysis of the links in ground truth structures indicating a maximal correlation in unlagged data. This may represent a limitation of the simplistic single-neuron models utilised here which, although utilising conductance based synapses to model prolonged effects of neurotransmitter release, did not include an explicit synaptic delay. This may explain the poor performance of all methods on these data. With an extremely large number of observations, structure learning becomes slower, presenting the challenging problem of balancing the length of recordings with temporal resolution. It may be possible to increase temporal resolution whilst maintaining recording length when learning a single structure, as opposed to the hundreds of repeated learning tasks here. However, the findings of Eldawlatly et al. (2010) suggest that reducing the Markov lag below the true synaptic lag causes a much more severe reduction in performance than observed here. Additionally, our supplementary analysis surrounding the number of data-points used in structure learning suggested that care must also be taken to avoid over-fitting when increasing length of data (SI 5). In the neural-population model, cross-correlation analysis of existing connections gave a median optimal lag of 1 ms and a mode of 2 ms. The comparability of these values to the resolution of neural-population data used in structure learning likely contributed to the increased performance of discrete DBNs and MVGC compared to the single-neuron data. Interestingly, there does not appear to be a benefit to continuous DBNs, which over-fit neural-population much more severely than single-neuron data. Future work may focus on the trade off between data length and resolution in these methods.

Given discrete DBNs’ consistency across data types relative to MVGC, future work may focus on development of these metrics to further improve their performance, providing a versatile and reliable tool for NIF structure learning. Given the number of structures learned in this paper, a maximum Markov lag of 1 was utilised throughout analysis. Future work may focus on quantifying the benefits of increasing this value for data with larger and more variable true lags, such as the neural-population data utilised here. Additionally, the iss parameter utilised in BDe may be increased to encourage this method to learn an increased number of connections (Steck & Jaakkola, 2002; Ueno, 2010). This may improve the fit of models learned using this score, encouraging BDe to produce networks with increased recall. Previous results suggest this effect may be especially beneficial in large datasets such as those available in neuroscience (Ke et al., 2025; Ulusoy & Geduk, 2024). It is likely, however that this increase in recall will be accompanied by a decrease in precision, and further work is required to investigate how increasing this parameter changes the positioning of BDe in precision recall space. Another benefit of DBNs comes from the ability to incorporate structural information into the scoring process. This was not tested here due to the nature of the simulation meaning information flow is defined by the structural connectivity. However, there has been some success in combining information from fMRI and diffusion tensor imaging in the structure learning process (Dang et al., 2018). This may be especially beneficial for single cell data from organisms such as *Drosophila*, where the structural connectome is well characterised (Azevedo et al., 2024; Dorkenwald et al., 2024; Takemura et al., 2024; Winding et al., 2023).

## Conclusion

This work has shown continuous DBNs to over-fit simulated single-neuron and neural-population data, to the extent that they perform close to chance on the latter data type. Discrete DBNs achieved a better balance, although tended to learn overly sparse networks. Discrete AIC score achieved the highest performance of all DBN scores, and the highest performance overall on single-neuron data. MVGC performed close to chance on single-neuron data, but achieved high performance on neural-population data when utilising significance testing over connection presence. This same significance testing depleted performance on single-neuron data. Taken together these findings illustrate the tendency of continuous DBNs to learn much denser structures than their discrete counterparts on neuronal data, the importance of considering data type when selecting structure learning methods, and the ambiguity around correct statistical testing measures when utilising MVGC. Overall, discrete DBNs should be used when learning NIF structure in highly non-linear data, whereas MVGC with significance testing should be used for more linear neuronal-population data. Future work may focus on optimising discrete DBNs for use on neuronal data, potentially developing them into a versatile tool across neuronal data types.

## Supplementary Information

Below is the link to the electronic supplementary material.

Supplementary file 1 (pdf 1824 KB)

## Data Availability

All scripts used in this study are available at: https://github.com/jacobhegarty/DBN-scores-neuro. This includes use of the R package DBayesNet, which was developed alongside this work and is available at https://github.com/jacobhegarty/DBayesNet.
