# Supplementary material for: Dynamic Bayesian networks for neural information flow: evaluation of continuous and discrete scoring metrics
Source: J Comput Neurosci. 2026 Jul 28;54(4):577–98. doi: 10.1007/s10827-026-00945-6 (PMC13710630; doi:10.1007/s10827-026-00945-6)
Supplement: Supplementary file 1 [file 10827_2026_945_MOESM1_ESM.pdf]

Dynamic Bayesian networks for neural information flow: evaluation of continuous and discrete scoring metrics

Supplementary information

1 Level of observation noise

The effect of increasing observation-level noise on structure learning performance was tested via addition of white noise to traces at varying signal to noise ratios (SNR). The variance of noise to be added was calculated at the node level to produce an SNR of 10, 8, 4, 2 and 1. In the results presented below these values are expressed as their inverse, giving the original analysis with no observation-level noise an intuitive value of 0. The first 10 simulations from both single-neuron and neural-population data were tested.

In single-neuron data, the relative positioning of all methods in precision-recall space remains fairly constant with increasing noise, with decreases in both precision and recall (Figure 1). Discrete DBNs suffer the largest decrease in precision, with a similar level to other methods at the largest SNR tested of 1 (Figure 1b).

In neural-population data, continuous DBNs and LASSO remain constant in performance, representing their strong over fit of the data (Figure 1). MVGC based methods suffer a large decrease in precision with increasing noise (Figure 1b). Discrete DBNs exhibit a small decrease in average recall, with relatively stable precision (Figure 1b,c). This promotes discrete DBNs as the most robust type of method for noisy neural-population data. However, further study with larger samples is required to verify this.

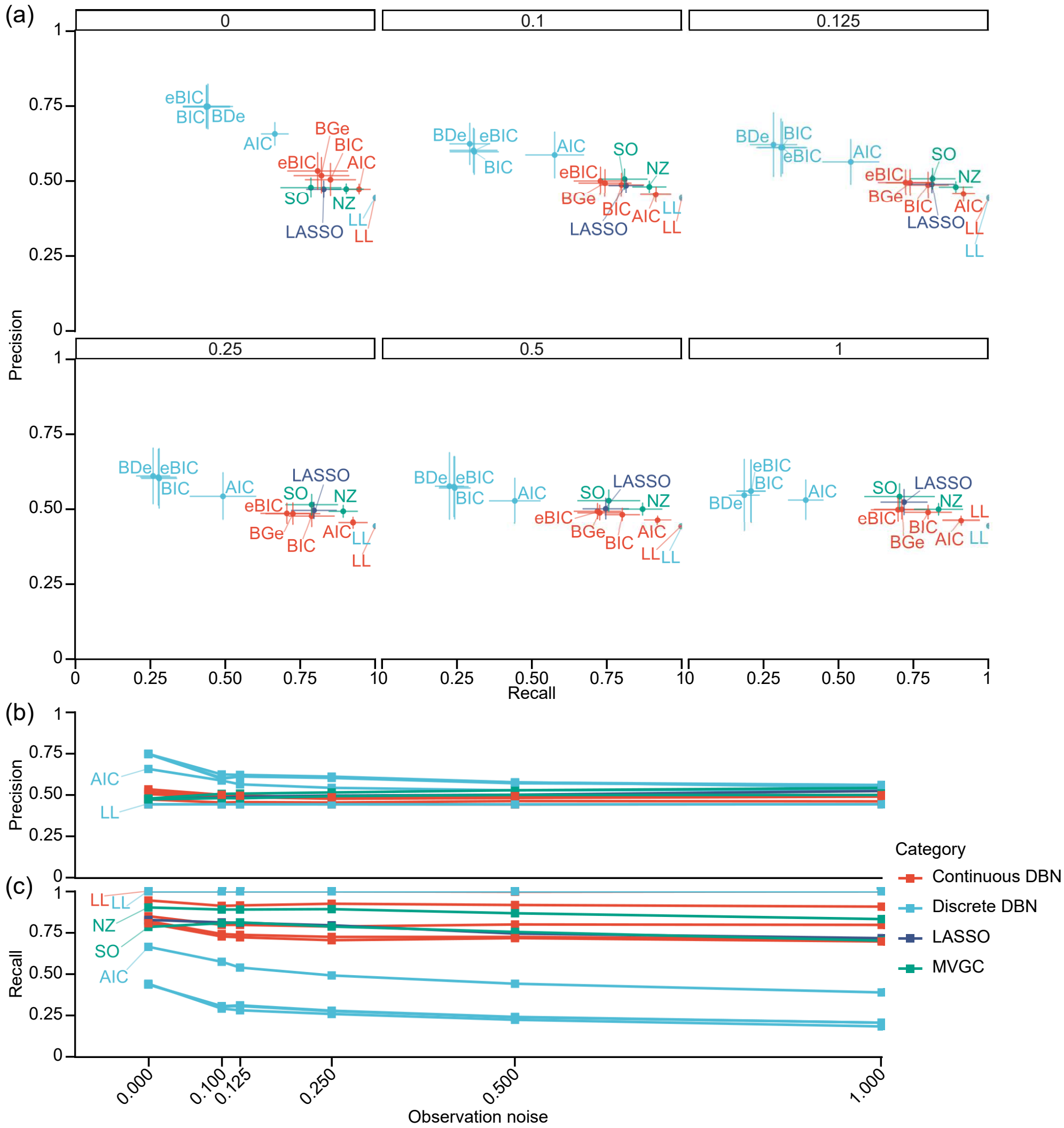

Fig. 1 Performance of structure learning methods on single-neuron data with increasing observation noise. Ten simulations were used, with white-noise added to each node to provide a noise to signal ratios from 0 to 1. a) Facets show average performance of DBN scores (eBIC = extended Bayesian information criterion, BIC = Bayesian information criterion, AIC = Akaike information criterion, LL = log-likelihood, BDe = Bayesian Dirichlet equivalent, BGe = Bayesian Gaussian equivalent), LASSO (purple) and MVGC (SO = Significant-only, NZ = Non-zero) for each group. Error-bars represent 1 SD either side of the mean. b) Average precision with increasing noise. c) Average recall with increasing noise. Only structure learning methods which separate from other methods in the category are labelled.

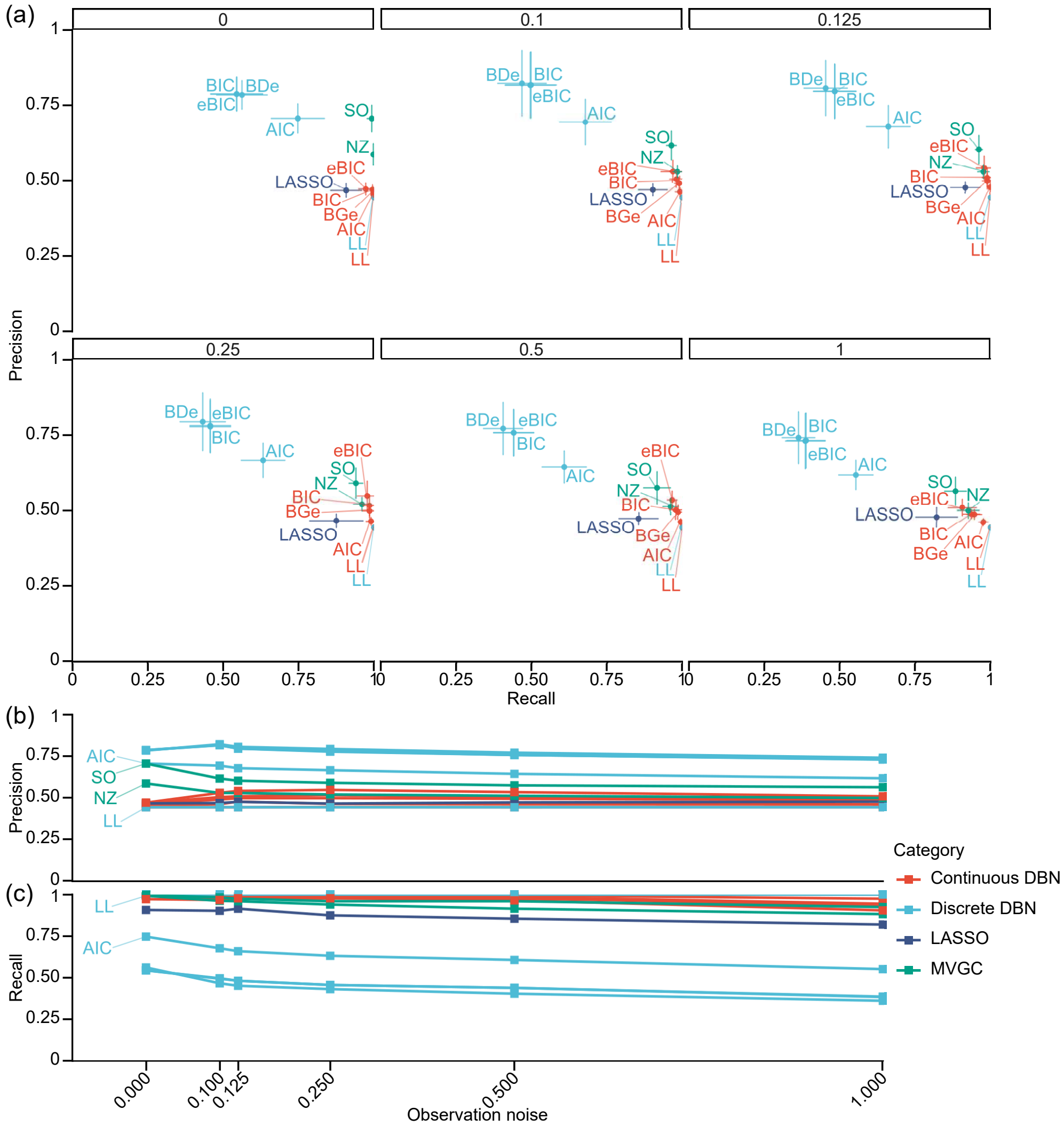

Fig. 2 Performance of structure learning methods on neural-population data with increasing observation noise. Ten simulations were used, with white-noise added to each node to provide a noise to signal ratios from 0 to 1. a) Facets show average performance of DBN scores (eBIC = extended Bayesian information criterion, BIC = Bayesian information criterion, AIC = Akaike information criterion, LL = log-likelihood, BDe = Bayesian Dirichlet equivalent, BGe = Bayesian Gaussian equivalent), LASSO (purple) and MVGC (SO = Significant-only, NZ = Non-zero) for each group. Error-bars represent 1 SD either side of the mean. b) Average precision with increasing noise. c) Average recall with increasing noise. Only structure learning methods which separate from other methods in the category are labelled.

2 Network size

The effect of network size on structure learning performance was investigated via simulation of additional single-neuron and neural-population datasets of both 20 and 50 nodes. Given increased computational complexity of learning the structure of networks of this size, only 10 simulations were performed for each group. The complete consistency of networks learned from each random restart in the hill-climb algorithm implies a simplistic solution space; as such, DBNs were learned here using a single hill-climb starting from an empty network. Further, given their tendency to learn fully connected networks, log-likelihood scores were not tested here. Other than this, all simulation and structure learning parameters remained constant. The following results compare the results on these datasets with the first 10 simulations from the original 10 node datasets, to provide consistency in sample size. In interpreting these findings it is important to note that increasing networks size reduces the chance level of performance.

In single neuron data, continuous DBNs, LASSO and MVGC decrease in precision in line with chance, with constant recall (Figure 3). In contrast, discrete DBNs do not exhibit the same decrease in precision. Other than this, relative ranking of all methods in precision-recall space remains constant with increasing network size (Figure 3). In neural-population data a similar effect is seen, although here the precision of significant-only does not fall in line with chance, with a slight increase for 20 to 50 node networks (Figure 4). These preliminary findings suggest that significance testing becomes both more necessary and effective in the MVGC paradigm when analysing larger networks. This effect may be related to the Bonferroni correction applied in significance testing, which scales with network size and warrants further investigation into the effectiveness of this correction at different network sizes. These findings also promote the robustness of discrete DBNs, especially for non-linear single-neuron data.

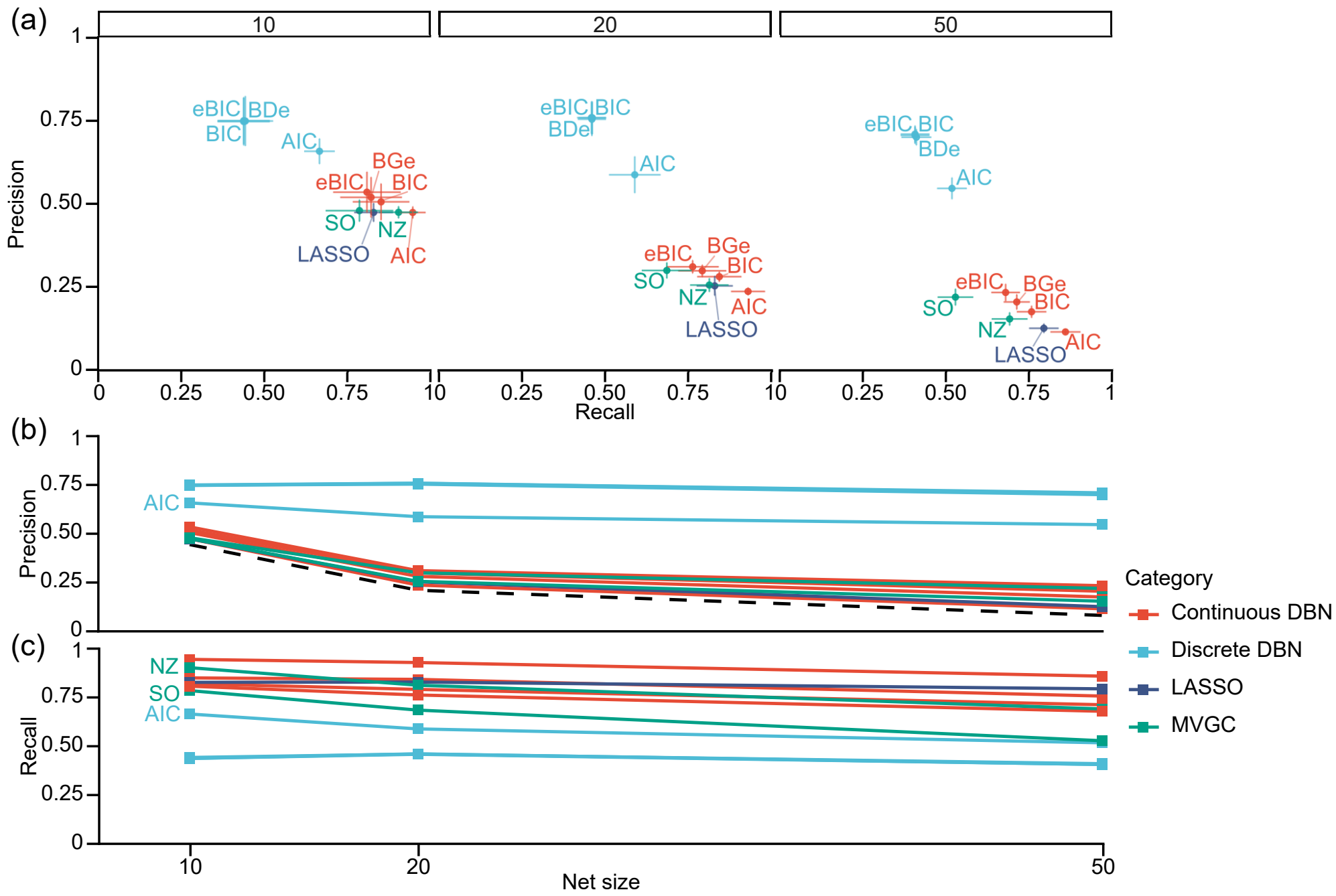

Fig. 3 Performance of structure learning methods on single-neuron data with increasing network size, as measured by out-degree of each node. Ten simulations each of networks with 10,20 and 50 neurons were tested. a) Facets show average performance of DBN scores (eBIC = extended Bayesian information criterion, BIC = Bayesian information criterion, AIC = Akaike information criterion, LL = log-likelihood, BDe = Bayesian Dirichlet equivalent, BGe = Bayesian Gaussian equivalent), LASSO (purple) and MVGC (SO = Significant-only, NZ = Non-zero) for each group. Error-bars represent 1 SD either side of the mean. b) Average precision with increasing network size. Dashed line represents expected precision if picking links at random. c) Average recall with increasing network size. Only structure learning methods which separate from other methods in the category are labelled.

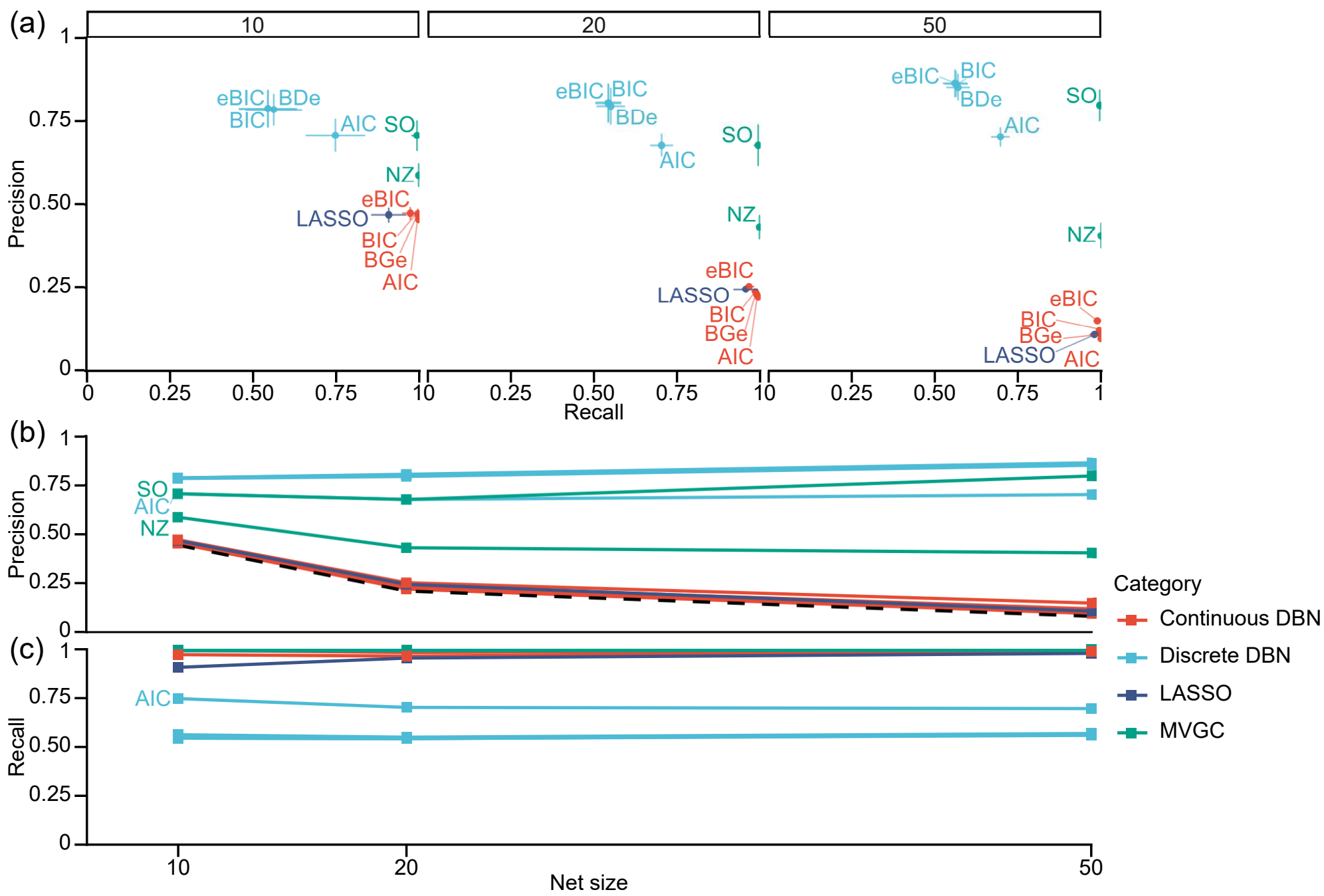

Fig. 4 Performance of structure learning methods on neural-population data with increasing network size, as measured by out-degree of each node. Ten simulations each of networks with 10,20 and 50 nodes were tested. a) Facets show average performance of DBN scores (eBIC = extended Bayesian information criterion, BIC = Bayesian information criterion, AIC = Akaike information criterion, LL = log-likelihood, BDe = Bayesian Dirichlet equivalent, BGe = Bayesian Gaussian equivalent), LASSO (purple) and MVGC (SO = Significant-only, NZ = Non-zero) for each group. Error-bars represent 1 SD either side of the mean. b) Average precision with increasing network size. Dashed line represents expected precision if picking links at random. c) Average recall with increasing network size. Only structure learning methods which separate from other methods in the category are labelled.

3 Network density

The effect of network density on structure learning performance was investigated by simulation of additional single-neuron and neural-population datasets with an out degree of 2 and 6, meaning each node sends either 2 or 6 connections to other randomly selected nodes respectively. Each group consisted of 10 simulations. Simulation and structure learning was performed in the same manner as the main analysis, comparing sparse and dense network findings to the first 10 simulations from the main analysis. In interpreting these findings it is important to note that increasing network density increases the level of precision which could be achieved by chance.

In single-neuron simulations, the positioning of structure learning methods relative to each other remains mostly constant across all network densities tested (Figure 5a). Precision of all methods increased with increasing network density, although not at the same rate as chance (Figure 5b). Recall of discrete DBNs had an inverse relationship with network density, whereas continuous DBNs, MVGC and LASSO maintain a high recall attributable to their pattern of over-fitting (Figure 5c). Interestingly, in sparser networks, discrete AIC achieves similar recall to continuous DBNs, MVGC and LASSO, further promoting it as the most robust method for single-neuron data.

In neural-population simulations, a similar effect is seen, with relative ranking of methods remaining constant (Figure 6a). Precision of continuous DBN scores and LASSO increase with network density in line with chance, remaining at a constant high recall characteristic of their over-fitting (Figure 6b,c). The precision of discrete DBN scores increase slightly with increasing network density, accompanied with a decrease in recall, demonstrating the increased under-fitting of these methods (Figure 6b,c). Precision of both significant-only and non-zero MVGC remains approximately constant, meaning the precision of these methods is approaching chance for networks with the highest out degree of 6 (Figure 6b). The recall of these methods remains high at all densities (Figure 6c). The lack of improvement in the precision of MVGC based methods in line with chance further promotes discrete DBNs as a more robust method across network structures.

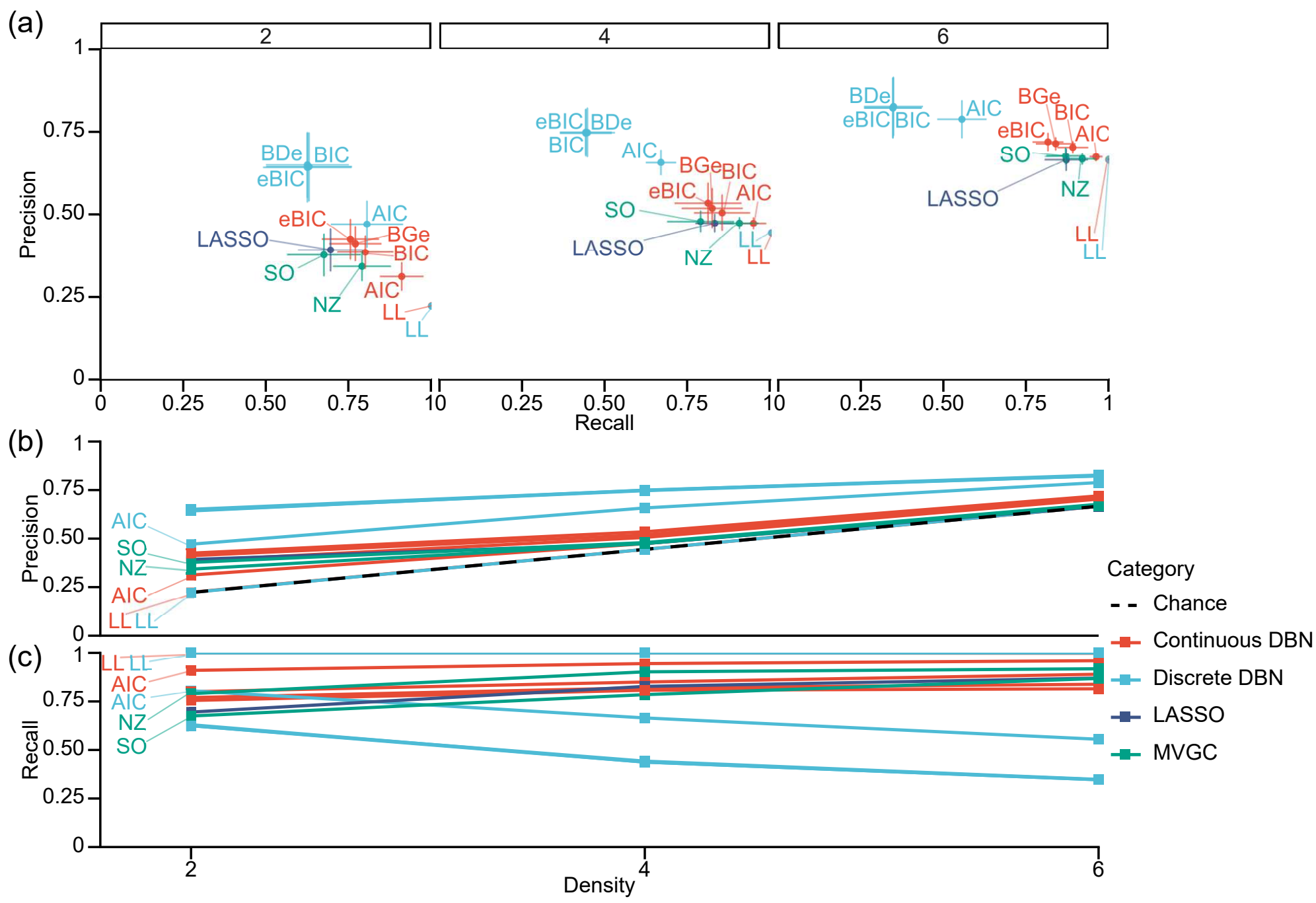

Fig. 5 Performance of structure learning methods on single-neuron data with increasing network density, as measured by out-degree of each node. Ten simulations each of networks with out-degrees of 2, 4 and 6 were tested. a) Facets show average performance of DBN scores (eBIC = extended Bayesian information criterion, BIC = Bayesian information criterion, AIC = Akaike information criterion, LL = log-likelihood, BDe = Bayesian Dirichlet equivalent, BGe = Bayesian Gaussian equivalent), LASSO (purple) and MVGC (SO = Significant-only, NZ = Non-zero) for each group. Error-bars represent 1 SD either side of the mean. b) Average precision with increasing network density. Dashed line represents expected precision if picking links at random. c) Average recall with increasing network density. Only structure learning methods which separate from other methods in the category are labelled.

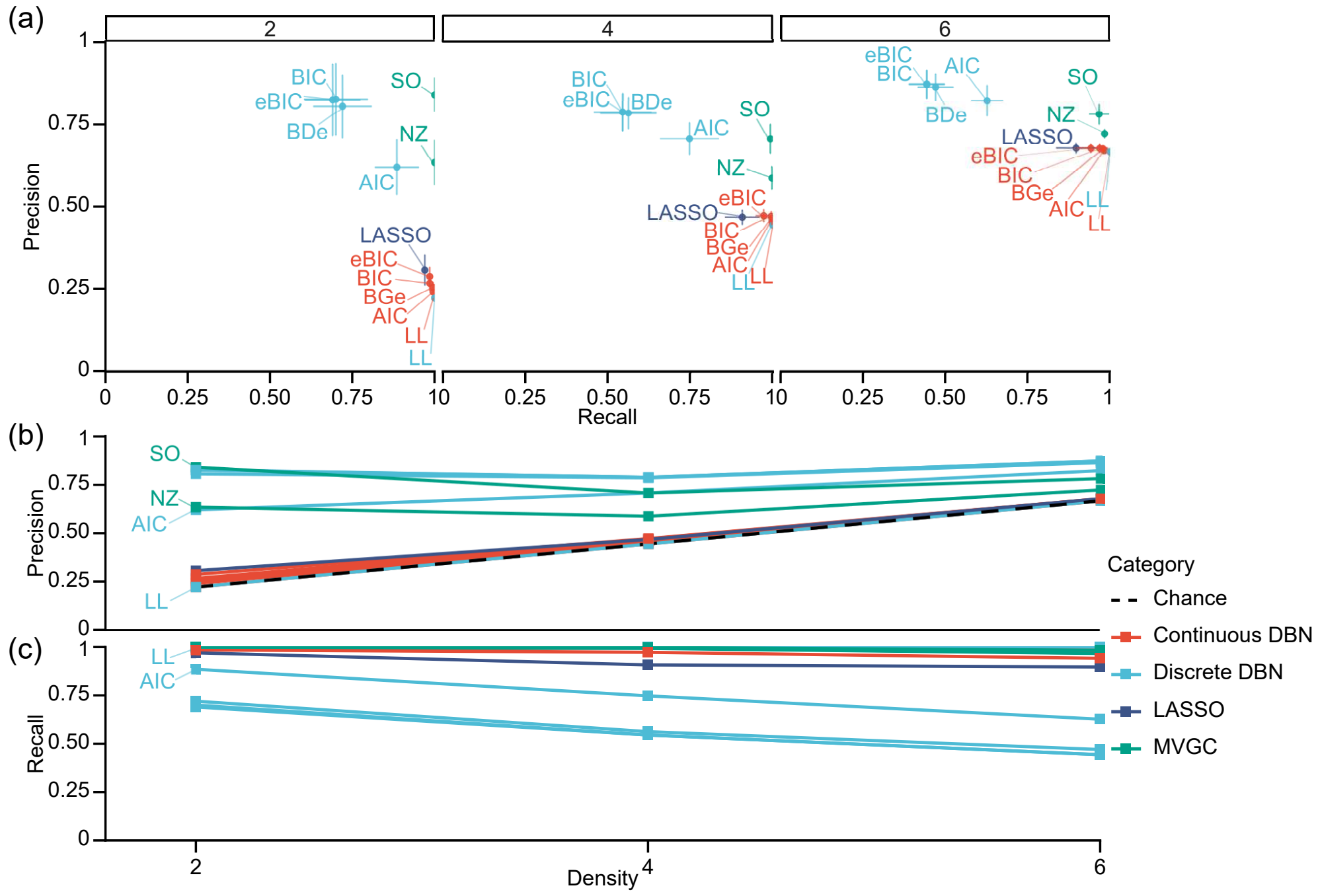

Fig. 6 Performance of structure learning methods on neural-population data with increasing network density, as measured by out-degree of each node. Ten simulations each of networks with out-degrees of 2, 4 and 6 were tested. a) Facets show average performance of DBN scores (eBIC = extended Bayesian information criterion, BIC = Bayesian information criterion, AIC = Akaike information criterion, LL = log-likelihood, BDe = Bayesian Dirichlet equivalent, BGe = Bayesian Gaussian equivalent), LASSO (purple) and MVGC (SO = Significant-only, NZ = Non-zero) for each group. Error-bars represent 1 SD either side of the mean. b) Average precision with increasing network density. Dashed line represents expected precision if picking links at random. c) Average recall with increasing network density. Only structure learning methods which separate from other methods in the category are labelled.

4 Level of inhibition

The effect of levels of inhibition in the neuronal network simulation on structure learning performance was investigated in networks with 10%, 30%, 50% and 70% inhibition. New random network structures were simulated with these proportions of inhibitory neurons in single-neuron simulations and inhibitory connections in neural-population simulations. All groups contained 10 simulations, with the first 10 simulations from the main analysis datasets being used for the 30% single-neuron and 50% neural-population groups.

In single-neuron data the relative positioning of all methods in precision recall space remain constant other than a slight decrease in recall of discrete DBNs and MVGC with increasing inhibition (Figure 7). In neural-population data, recall of discrete DBNs increase from networks with a 0.1 to a 0.3 proportion of inhibitory connections, but decrease slightly with increasing inhibition beyond this (Figure 8). Precision increases with level of inhibition for these scores. In MVGC, an initial decrease followed by an increase in precision and gradually increasing recall with increasing inhibition is observed. LASSO achieves a relatively high precision at 0.1 inhibitory proportion, before decreasing as inhibition increases. The performance of continuous DBNs appear to remain fairly constant with increasing inhibition, other than an increase in recall from 0.1 to 0.3 inhibitory proportion. Taken together, these results highlight a dependence of discrete DBNs, MVGC and LASSO on network inhibition.

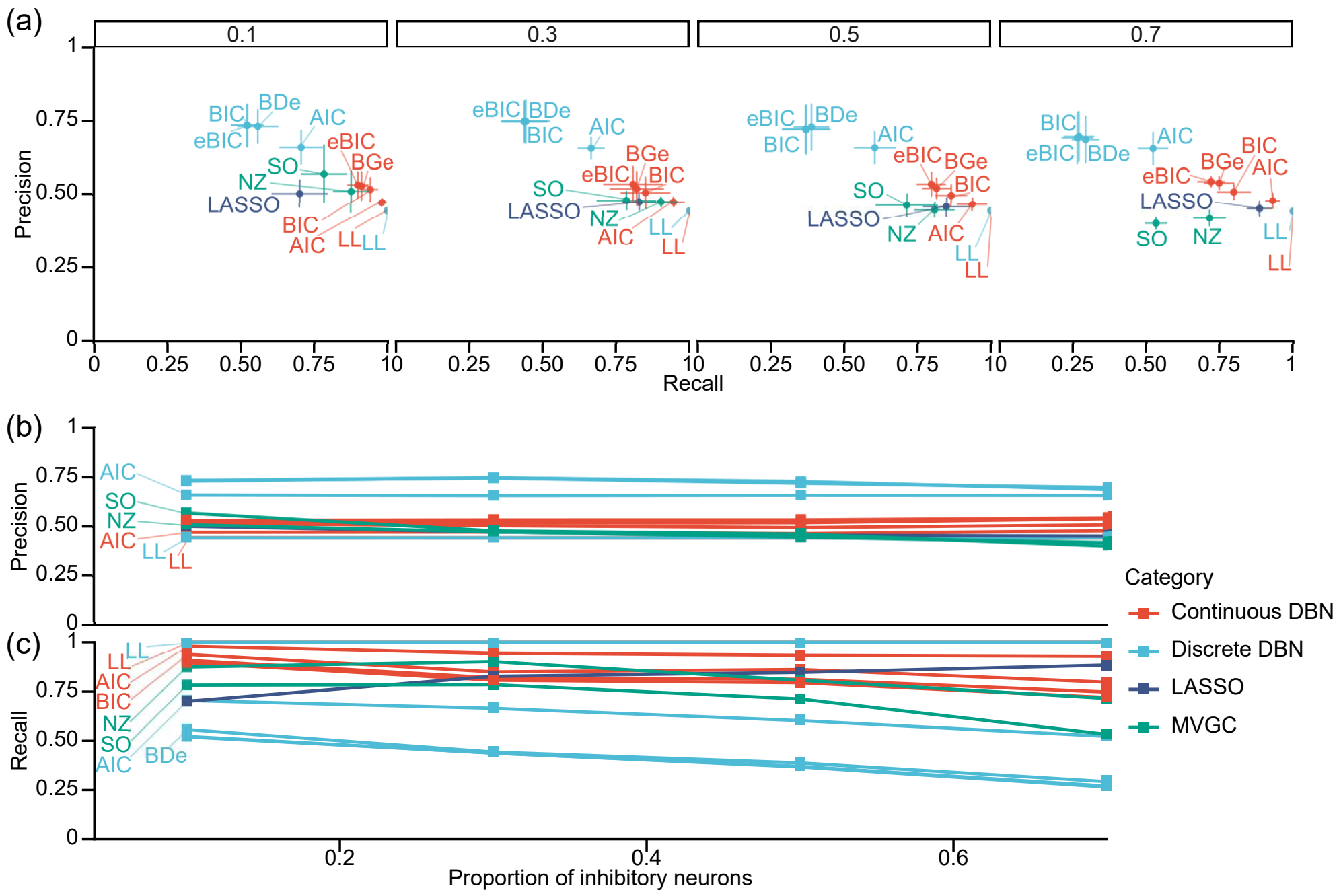

Fig. 7 Performance of structure learning methods on single-neuron data with increasing proportion of inhibitory neurons. Ten networks of 10%, 30%, 50% and 70% inhibitory neurons were tested. a) Facets show average performance of DBN scores (eBIC = extended Bayesian information criterion, BIC = Bayesian information criterion, AIC = Akaike information criterion, LL = log-likelihood, BDe = Bayesian Dirichlet equivalent, BGe = Bayesian Gaussian equivalent), LASSO (purple) and MVGC (SO = Significant-only, NZ = Non-zero) for each group. Error-bars represent 1 SD either side of the mean. b) Average precision with increasing inhibition. c) Average recall with increasing inhibition. Only structure learning methods which separate from other methods in the category are labelled.

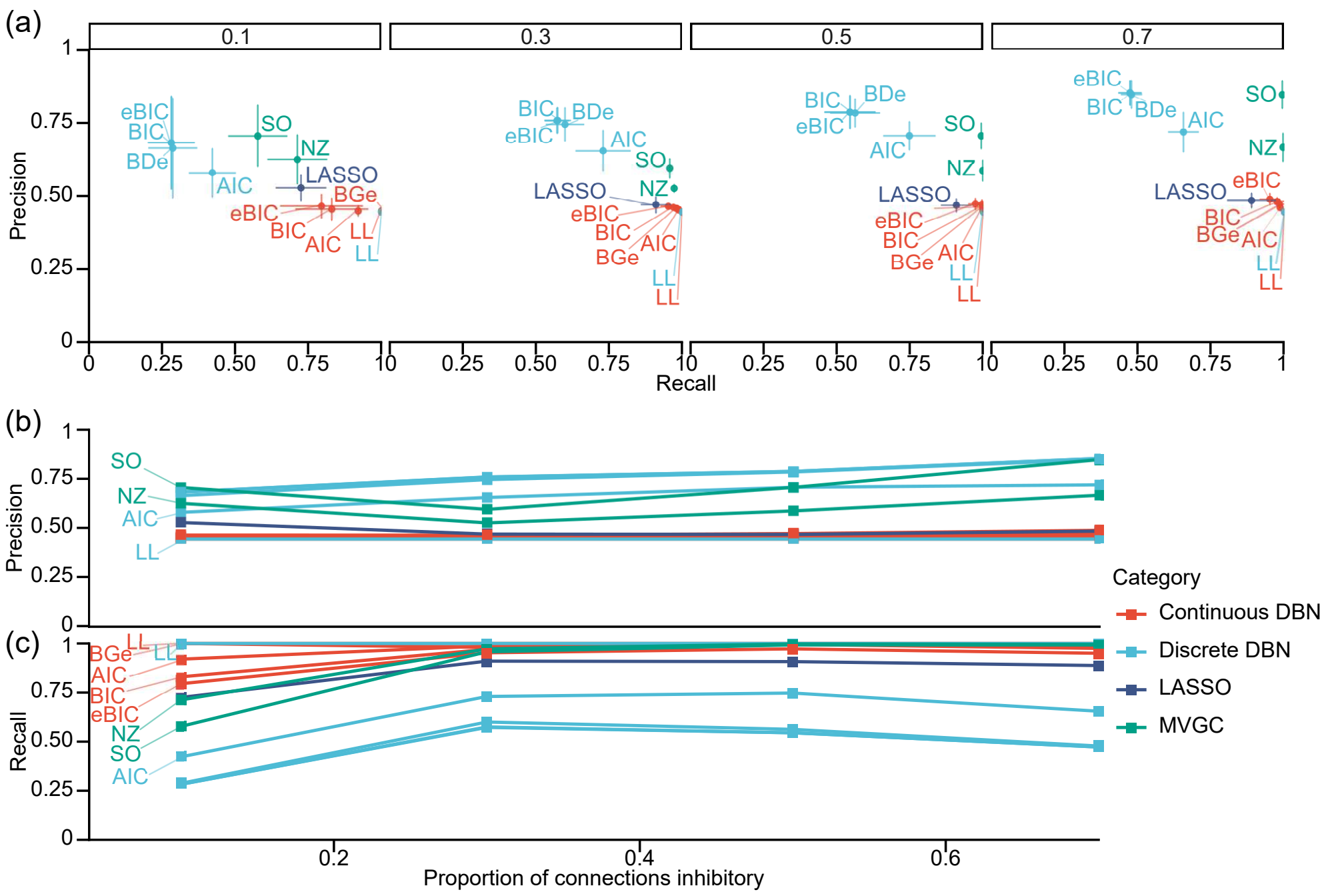

Fig. 8 Performance of structure learning methods on neural-population data with increasing proportion of inhibitory connections. Ten networks of 10%, 30%, 50% and 70% inhibitory connections were tested. a) Facets show average performance of DBN scores (eBIC = extended Bayesian information criterion, BIC = Bayesian information criterion, AIC = Akaike information criterion, LL = log-likelihood, BDe = Bayesian Dirichlet equivalent, BGe = Bayesian Gaussian equivalent), LASSO (purple) and MVGC (SO = Significant-only, NZ = Non-zero) for each group. Error-bars represent 1 SD either side of the mean. b) Average precision with increasing inhibition. c) Average recall with increasing inhibition. Only structure learning methods which separate from other methods in the category are labelled.

5 Number of data points

The effect of number of data points on structure learning performance was tested via subsampling of original datasets to 100, 1000 and 10 000 data points. Data resolution remained the same, meaning that this variation represents change in both amount of data to be represented by structure learning methods and length of simulation. The first 10 simulations from both single-neuron and neural-population data were tested for the effects of data length.

In single-neuron data, variance in performance decreases with increasing data length. At 100 and 1000 data points, LASSO and continuous AIC may be preferable methods due to their reduced standard deviation in the precision dimension, however performance is highly variable across all methods (Figure 9a). Comparing average precision, MVGC and most discrete DBNs increase from 100 to 10 000 data points, after which precision plateaus, whereas other methods remain constant (Figure 9b). Recall of all methods other than log-likelihood scores appears to increase with more data (Figure 9c). The lack of improvement in precision of continuous scores and LASSO with increasing availability of information suggests that these methods are not extracting information on network structure effectively, with their increased recall merely representing increased density in learned networks as data length increases.

In neural-population data, there is a similar increase in variance with less data, although this is predominately seen in discrete AIC and BDe scores in the precision domain (Figure 10a). At 100 data points, discrete eBIC and BIC methods do not learn any connections in any of the tested simulations. Similarly, BDe and AIC perform with high average precision and low recall at low data availability. This may be indicative of under-fitting, whereby these methods learn few, or occasionally no, connections, but these connections are accurate. Precision peaks for all discrete DBNs at 1000 data points, beyond which is gradually reduces. This is accompanied by an increase in recall, demonstrating a less severe over-fit of with increased data. Both MVGC methods follow a similar pattern, however they reach an extremely high level of performance at 10 000 data points, with an average recall of 0.872 and precision of 0.940 for significant-only networks. At 100 000 data points there is a large reduction in the precision of these methods, with only a moderate increase in recall. This suggests that MVGC reaches an optimal fit of this data at around 10 000 data points, beyond which it begins to over-fit. Future work may focus on the optimal data lengths for MVGC, with a goal of avoiding this over-fitting effect. Continuous DBNs and LASSO perform at close to chance precision at all data lengths, with increases in recall further suggesting an increased tendency of these methods to learn more densely connected networks with more data.

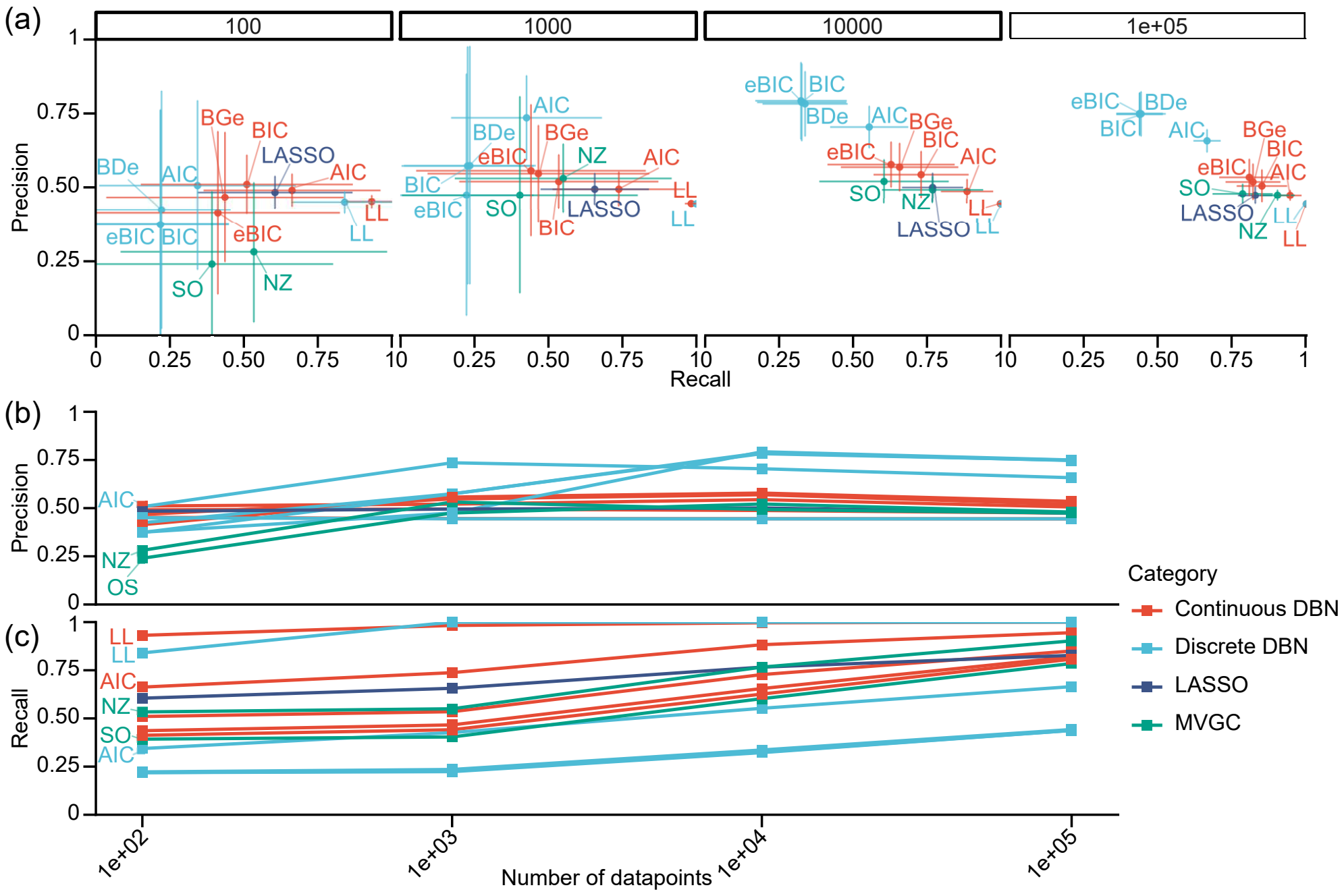

Fig. 9 Performance of structure learning methods on single-neuron data with an increasing number of data points. Ten simulations were used, with 100, 1000, 10 000 and 100 000 data points supplied to each structure learning algorithm. a) Facets show average performance of DBN scores (eBIC = extended Bayesian information criterion, BIC = Bayesian information criterion, AIC = Akaike information criterion, LL = log-likelihood, BDe = Bayesian Dirichlet equivalent, BGe = Bayesian Gaussian equivalent), LASSO (purple) and MVGC (SO = Significant-only, NZ = Non-zero) for each group. Error-bars represent 1 SD either side of the mean. b) Average precision with increasing number of data points. c) Average recall with increasing number of data points. Only structure learning methods which separate from other methods in the category are labelled.

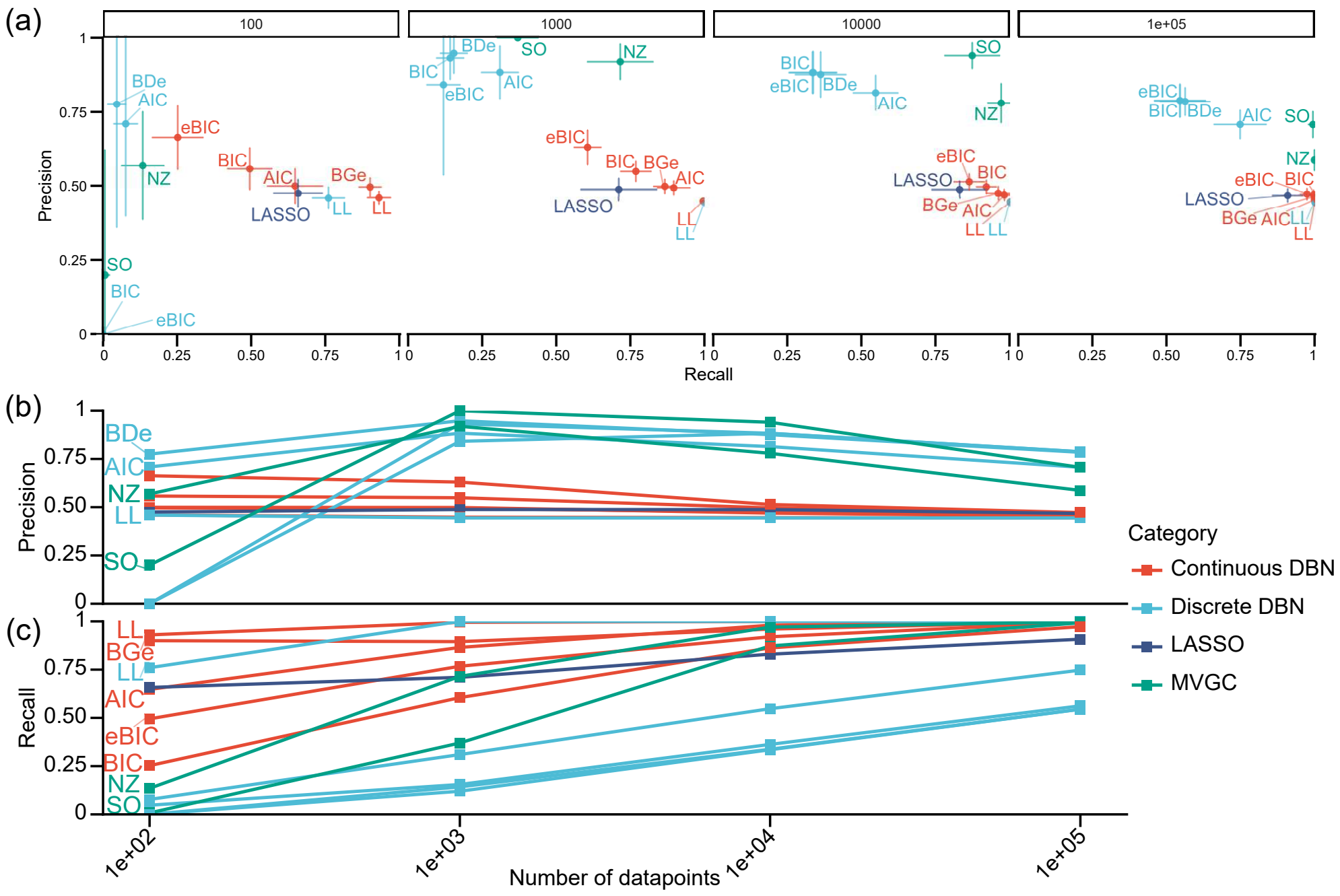

Fig. 10 Performance of structure learning methods on neural-population data with an increasing number of data points. Ten simulations were used, with 100, 1000, 10 000 and 100 000 data points supplied to each structure learning algorithm. a) Facets show average performance of DBN scores (eBIC = extended Bayesian information criterion, BIC = Bayesian information criterion, AIC = Akaike information criterion, LL = log-likelihood, BDe = Bayesian Dirichlet equivalent, BGe = Bayesian Gaussian equivalent), LASSO (purple) and MVGC (SO = Significant-only, NZ = Non-zero) for each group. Error-bars represent 1 SD either side of the mean. b) Average precision with increasing number of data points. c) Average recall with increasing number of data points. Only structure learning methods which separate from other methods in the category are labelled.
